# TRIC-A shapes oscillatory Ca^2+^ signals by interaction with STIM1/Orai1 complexes

**DOI:** 10.1371/journal.pbio.3000700

**Published:** 2020-04-24

**Authors:** Niroj Shrestha, Bernadett Bacsa, Hwei Ling Ong, Susanne Scheruebel, Helmut Bischof, Roland Malli, Indu Suresh Ambudkar, Klaus Groschner

**Affiliations:** 1 Gottfried Schatz Research Center-Biophysics, Medical University of Graz, Graz, Austria; 2 Secretory Physiology Section, NIDCR, NIH, Bethesda, Maryland, United States of America; 3 Gottfried Schatz Research Center-Molecular Biology and Biochemistry, Medical University of Graz, Graz, Austria; University of Padova, ITALY

## Abstract

Trimeric intracellular cation (TRIC) channels have been proposed to modulate Ca^2+^ release from the endoplasmic reticulum (ER) and determine oscillatory Ca^2+^ signals. Here, we report that TRIC-A–mediated amplitude and frequency modulation of ryanodine receptor 2 (RyR2)-mediated Ca^2+^ oscillations and inositol 1,4,5-triphosphate receptor (IP_3_R)-induced cytosolic signals is based on attenuating store-operated Ca^2+^ entry (SOCE). Further, TRIC-A–dependent delay in ER Ca^2+^ store refilling contributes to shaping the pattern of Ca^2+^ oscillations. Upon ER Ca^2+^ depletion, TRIC-A clusters with stromal interaction molecule 1 (STIM1) and Ca^2+^-release–activated Ca^2+^ channel 1 (Orai1) within ER–plasma membrane (PM) junctions and impairs assembly of the STIM1/Orai1 complex, causing a decrease in Orai1-mediated Ca^2+^ current and SOCE. Together, our findings demonstrate that TRIC-A is a negative regulator of STIM1/Orai1 function. Thus, aberrant SOCE could contribute to muscle disorders associated with loss of TRIC-A.

## Abstract 2

Trimeric intracellular cation (TRIC) channels have been proposed to modulate calcium release from the endoplasmic reticulum (ER) and to determine oscillatory calcium signals. This study shows that upon depletion of ER calcium, TRIC-A coclusters and interferes with STIM1-Orai1 coupling within ER-plasma membrane junctions.

## Introduction

Trimeric intracellular cation (TRIC) channels have been identified as modulators of the sarco/endoplasmic reticulum (SR/ER) Ca^2+^ release mediated by either ryanodine receptors (RyRs) or inositol 1,4,5-trisphosphate receptors (IP_3_Rs). Both TRIC isoforms (TRIC-A and TRIC-B), also known as transmembrane protein 38A (TMEM38A) and TMEM38B, respectively, are ER-resident proteins with monovalent cation permeability [1]. Crystal structures of nonmammalian TRIC homologs revealed homotrimeric complexes harboring lipids and featuring an hourglass-shaped, hydrophilic pore within each of the 7-transmembrane domain subunits [2,3]. At rest, cation permeation in avian TRIC-A appears occluded by a highly conserved lysine residue within a voltage-sensing domain that is stabilized by Ca^2+^ binding to the luminal surface of the channel. Dissociation of Ca^2+^ from TRIC-A was shown to initiate a conformational transition in the voltage-sensing domain to move the occluding lysine and open the pore for counterion flux [4].

Both isoforms appear to provide complementary Ca^2+^ signaling functions in embryonic cardiomyocytes because double knockout of these gene products is lethal because of embryonic heart failure, characterized by diminished spontaneous Ca^2+^ transients and Ca^2+^ oxalate deposition in swollen SR [1]. TRIC-A^−/−^ mice are viable but exhibit abnormal SR Ca^2+^ mobilization in excitable muscles. While TRIC-A ablation inhibits RyR-mediated Ca^2+^ sparks in vascular smooth muscles, it also simultaneously enhances IP_3_R-mediated Ca^2+^ waves and oscillations due to SR Ca^2+^ overload, resulting in hypertension [5]. Skeletal muscles of TRIC-A^−/−^ mice display compromised Ca^2+^ sparks, slower voltage-induced Ca^2+^ release via RyR1, and irregular contractile force (mechanical alternans) during fatigue [1,6]. The phenotypes of TRIC-A^−/−^ observed in muscle tissues have been attributed to the lack of critical K^+^ countercurrent via the trimeric channel during SR Ca^2+^ release. However, replacing cytosolic K^+^ with Na^+^ or Cs^+^ in isolated cardiac SR microsomes or saponin-permeabilized myocytes failed to affect single RyR2 channel currents, open probability, and Ca^2+^ sparks. Moreover, RyRs have been proposed to mediate sufficient counter K^+^/Mg^2+^ flux, based on their nonselective permeability, to sustain Ca^2+^ release [7]. Hence, TRIC-A may not represent an indispensable prerequisite for efficient SR Ca^2+^ release as presumed earlier but still contribute to K^+^ equilibrium across SR and, in turn, restore membrane potential near 0 mV upon closure of RyRs during repetitive cycles of release events. Recently, a cardiac SR compartment model suggests SR Ca^2+^ release relies on a cascading network of ion permeabilities, including TRICs, to provide countercurrents and establish ion homeostasis [8].

Store-operated Ca^2+^ entry (SOCE) has been reported as a critical determinant of IP_3_R/RyR-induced oscillatory Ca^2+^ signals in many cell types, including muscle and immune cells [9–13]. In the absence of Ca^2+^ entry, refilling of ER Ca^2+^ stores is attenuated along with a rundown of oscillatory Ca^2+^ signals. SOCE is mediated by stromal interaction molecule 1 (STIM1) and Ca^2+^-release–activated Ca^2+^ channel 1 (Orai1), which form Ca^2+^-entry–competent complexes in response to a decrease in ER luminal Ca^2+^ ([Ca^2+^]_ER_). STIM1 is an ER-resident Ca^2+^ sensor for [Ca^2+^]_ER_ with its N-terminal EF-hand bound to [Ca^2+^]_ER_. Following [Ca^2+^]_ER_ depletion, Ca^2+^ dissociates from the EF-hand and causes conformational changes in STIM1 that result in oligomerization and translocation of the protein to ER–plasma membrane (PM) junctions [14–16]. Within ER–PM junctions, STIM1 clusters to recruit and activate the PM-resident Orai1 channel [17–20]. Subsequent Ca^2+^ entry via Orai1 causes sustained elevation of intracellular Ca^2+^ ([Ca^2+^]_i_) in cells where [Ca^2+^]_ER_ depletion is substantial, i.e., those stimulated with high [agonist]. Alternatively, in cells stimulated with relatively low [agonist], SOCE drives sustained Ca^2+^ oscillations, which are variable in frequency and linked specifically to downstream signaling pathways [13,21,22].

A number of previous studies have identified TRIC-A as a unique, muscle-specific [Ca^2+^]_ER_ sensing cation channel involved in facilitating [Ca^2+^]_ER_ release and regulating intracellular K^+^/Ca^2+^ homeostasis. How the intracellular channels modulate oscillatory Ca^2+^ signals is as yet incompletely understood. Here, we explored the function of TRIC-A by heterologous expression in human embryonic kidney 293 (HEK293) cells engineered to reconstitute muscle-type oscillatory Ca^2+^ cycling. This system enables investigation of TRIC-A function in the absence of endogenous background expression of this isoform. We provide evidence that TRIC-A modulates SOCE by affecting STIM1/Orai1 complex assembly and function, as well as ER Ca^2+^ store refilling. We show that TRIC-A channels cocluster with the STIM1/Orai1 Ca^2+^ signaling complex within ER–PM junctions upon ER Ca^2+^ store depletion. Coassembly of TRIC-A with STIM1/Orai1 attenuates STIM1/Orai1 interaction and SOCE-driven Ca^2+^ oscillations. Hence, we propose a novel, to our knowledge, function for TRIC-A as a modulator of SOCE and oscillatory Ca^2+^ signals. Aberrant SOCE and cellular Ca^2+^ handling could underlie the muscle disorders associated with loss of TRIC-A.

## Results

### TRIC-A modifies the frequency and amplitude of RyR2-mediated oscillations

**Fig 1:**
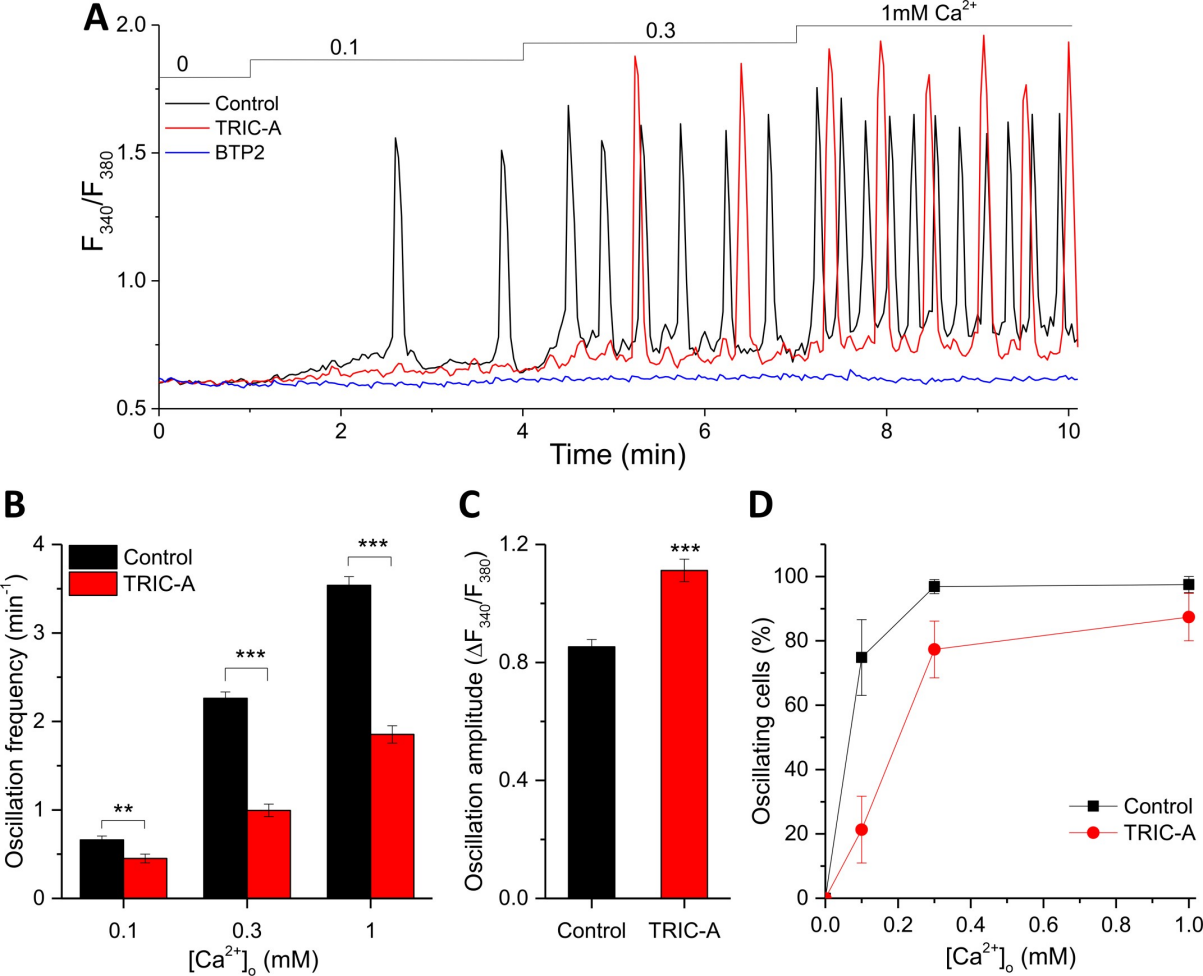
TRIC-A modifies the frequency and amplitude of RyR2-mediated cytosolic Ca^2+^ oscillations. (A) Traces of cytosolic Ca^2+^-sensitive Fura-2 ratio, representing SOICR-associated oscillations in mCherry-ER-3– (control, black) or TRIC-A-mCherry–transfected (TRIC-A, red) HEK293_RyR2 cell and lack of oscillations in 3 μM BTP2-incubated (BTP2, blue) control cell. (B) Ca^2+^ oscillation frequency at 0.1, 0.3, and 1 mM [Ca^2+^]_o_, (C) amplitude at 1 mM [Ca^2+^]_o_, and (D) proportion of oscillating cells in TRIC-A cells (*n* = 88) versus controls (*n* = 81); **p < 0.01, ***p < 0.001; mean ± SEM values are shown. Underlying data in panels (A–D) are included in S1 Data. BTP2, N-[4-[3,5-Bis(trifluoromethyl)pyrazol-1-yl]phenyl]-4-methylthiadizole-5-carboxamide; ER, endoplasmic reticulum; Fura-2, cytosolic Ca^2+^-sensitive fluorescent indicator; HEK293, human embryonic kidney 293; RyR, ryanodine receptor; SOICR, store-overload–induced Ca^2+^ release; TRIC, trimeric intracellular cation.

HEK293 cells stably expressing RyR2 (HEK293_RyR2 cells) offer an established model of relatively simple molecular composition to mimic the cardiac phenotype for studying RyR2-induced spontaneous Ca^2+^ oscillations, known as store-overload-induced Ca^2+^ release (SOICR) [23]. These oscillations occur because of SR Ca^2+^ overload in cardiac cells under various conditions, namely elevated extracellular Ca^2+^ ([Ca^2+^]_o_), ischemia/reperfusion, digitalis toxicity, and independently of membrane depolarization [24]. Raising [Ca^2+^]_o_ increases the steady-state ER Ca^2+^ content in HEK293_RyR2 cells, thereby promoting the discharge of the Ca^2+^ stores via RyR2, which are at the same time governed by local Ca^2+^ levels [23]. We first assessed the effect of TRIC-A on SOICR-associated oscillatory Ca^2+^ signals by transient expression of TRIC-A-mCherry in HEK293_RyR2; note that these cells do not express TRIC-A endogenously (S1A Fig). In S1B Fig, TRIC-A-mCherry exclusively colocalized with mCerulean-ER-5 (ER marker) and not with cyan fluorescent protein-glycosylphosphatidylinositol (CFP-GPI) (PM marker) (Pearson’s coefficient 0.97 ± 0.01 versus 0.25 ± 0.05). Cytosolic Ca^2+^ oscillations were recorded in response to [Ca^2+^]_o_ elevations from nominally free up to 1.0 mM (Fig 1A). As expected, the frequency of Ca^2+^ oscillations in HEK293_RyR2 cells positively correlated with [Ca^2+^]_o_. Expression of TRIC-A significantly reduced the frequency of oscillations at each [Ca^2+^]_o_ to about 50%–68% when compared to the respective controls (Fig 1B). This frequency modulation by TRIC-A was associated with a moderately but significantly enhanced average peak amplitude at 1 mM [Ca^2+^]_o_ (Fig 1C). Moreover, TRIC-A also significantly reduced the proportion of oscillating cells at each [Ca^2+^]_o_ (Fig 1D). To test whether the Ca^2+^ influx that mediates these cytosolic Ca^2+^ oscillations is via the SOCE pathway, HEK293_RyR2 cells were incubated with the SOCE inhibitor, N-[4-[3,5-Bis(trifluoromethyl)pyrazol-1-yl]phenyl]-4-methylthiadiazole-5-carboxamide (BTP2; 10 min) [25]. BTP2 indeed completely blocked these oscillations (Fig 1A), suggesting SOCE could be the major Ca^2+^ influx pathway for refilling ER Ca^2+^ stores and initiating Ca^2+^ discharge during the oscillatory cycle.

Besides cytosolic Ca^2+^ responses, we measured Ca^2+^ oscillations within the ER lumen using a genetically encoded ER-targeted Ca^2+^ sensor, D1ER [26] (S1C Fig). The frequency of Ca^2+^ oscillations within the ER lumen of HEK293_RyR2 cells was also positively correlated with increasing [Ca^2+^]_o_. Expression of TRIC-A-mCherry significantly reduced the frequency of ER Ca^2+^ signals by about 50% at each [Ca^2+^]_o_ as compared to controls (S1D Fig) while enhancing the average peak amplitude of individual ER Ca^2+^ store-depletion events (S1E Fig shows quantification at 1 mM [Ca^2+^]_o_). Moreover, TRIC-A clearly increased the time required to obtain complete ER Ca^2+^ store refilling (S1C Fig). Hence, the prominent phenotype of TRIC-A overexpression was decelerated ER Ca^2+^ refilling and profound prolongation of the period between discharge events. As noted in Fig 1A, SOICR-associated ER Ca^2+^ oscillations were also completely abrogated by treatment of cells with BTP2 (S1C Fig). Similarly, another classical inhibitor of SOCE, 2-Aminoethoxydiphenylborane (2-APB) (S2A and S2B Fig) or expression of a dominant negative Orai1 (E106Q) mutant [27] (S2C and S2D Fig) significantly reduced Ca^2+^ oscillation frequency upon addition of 0.1, 0.3, and 1 mM [Ca^2+^]_o_.

### TRIC-A attenuates SOCE irrespective of RyR2 expression

**Fig 2:**
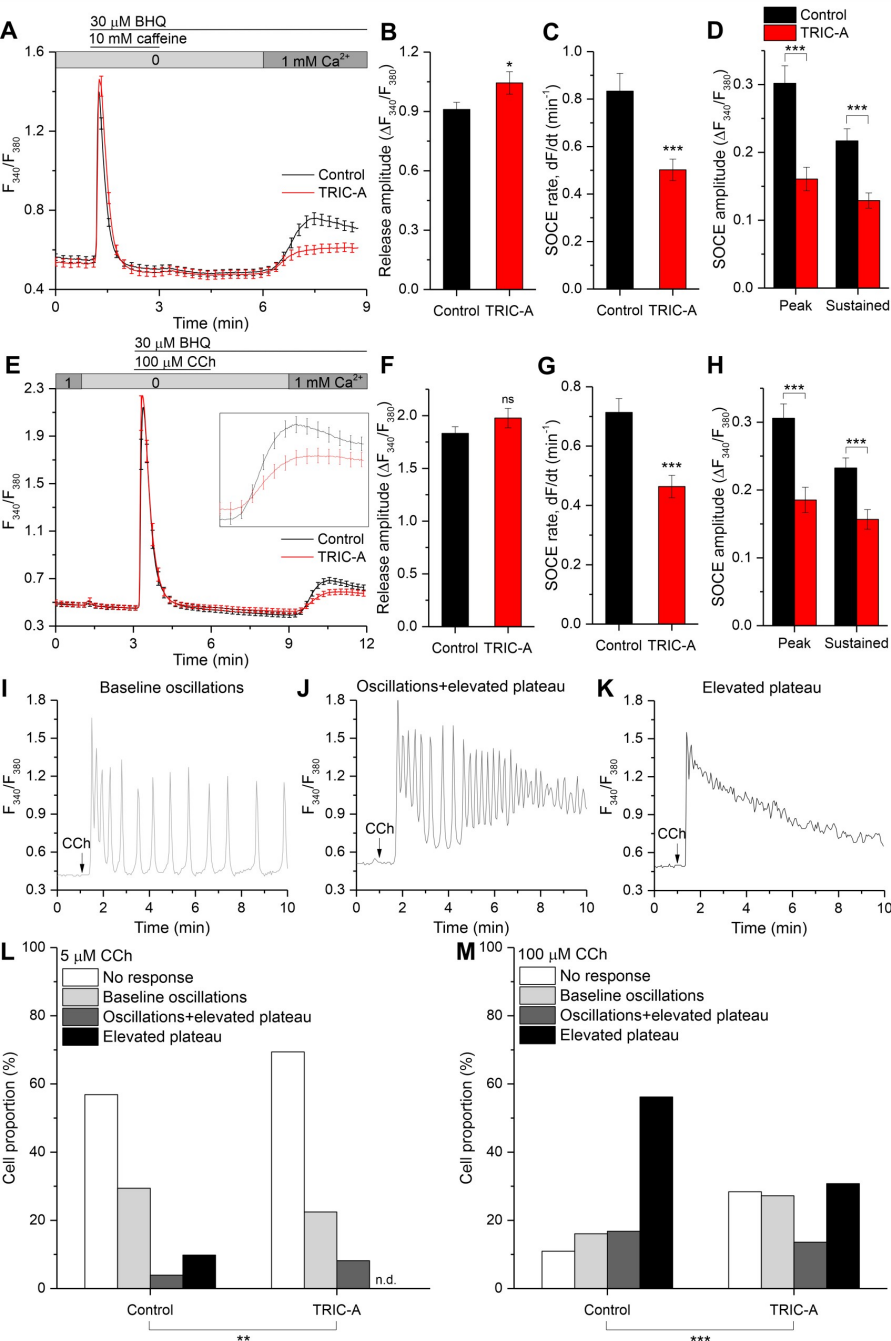
TRIC-A attenuates SOCE irrespective of RyR2 expression and dampens associated [Ca^2+^]_i_ responses to low and high stimuli levels. Average cytosolic Ca^2+^-sensitive Fura-2 traces in mCherry-ER-3 (control, black) or TRIC-A-mCherry (TRIC-A, red) transfected (A) HEK293_RyR2 cells and (E) wild-type HEK293 cells, showing SOCE after ER Ca^2+^ depletion with 10 mM caffeine + 30 μM BHQ and 100 μM CCh + 30 μM BHQ, respectively. Inset in (E) shows an enlarged phase of SOCE. Bar graphs show (B) ER Ca^2+^ release peak amplitude, (C) SOCE rate, and (D) peak and sustained SOCE amplitude in (A) TRIC-A (+) (*n* = 42) versus control (*n* = 49) HEK293_RyR2 cells. (F–H) show similar bar graphs as in (B–D) for (E) TRIC-A (+) (*n* = 30) versus control (*n* = 38) wild-type HEK293 cells. *p < 0.05, ***p < 0.001; mean values ± SEM are shown. (I–K) Representative traces showing various [Ca^2+^]_i_ responses in HEK293 cells stimulated with 5 or 100 μM CCh (arrow). Proportion (%) of cell population displaying various patterns of [Ca^2+^]_i_ response to (L) 5 μM and (M) 100 μM CCh. Overall patterns in TRIC-A (+) cells (*n* = 98 at 5 μM, *n* = 169 at 100 μM) were significantly different from that in controls (*n* = 102 at 5 μM, *n* = 137 at 100 μM); **p < 0.01,***p < 0.001; χ^2^ test. Underlying data in panels (A–M) are included in S1 Data. BHQ, 2,5-Di-*t*-butyl-1,4-benzohydroquinone; CCh, carbachol; ER, endoplasmic reticulum; Fura-2, cytosolic Ca^2+^-sensitive fluorescent indicator; HEK293, human embryonic kidney 293; ns, nonsignificant; RyR, ryanodine receptor; SOCE, store-operated Ca^2+^ entry; TRIC, trimeric intracellular cation.

Since our findings illustrated in Fig 1 and S1 Fig suggest that SOCE is involved in regulating SOICR in HEK293_RyR2 cells, we investigated whether TRIC-A modulates SOCE by a standard store-depletion/Ca^2+^ readdition protocol in HEK293_RyR2 cells. As shown in Fig 2A, ER Ca^2+^ was depleted by the RyR agonist, caffeine together with the sarco/endoplasmic reticulum Ca^2+^-ATPase (SERCA) inhibitor 2,5-Di-*t*-butyl-1,4-benzohydroquinone (BHQ), to prevent ER refilling in Ca^2+^-free external medium to measure Ca^2+^ release from ER, followed by readdition of 1 mM CaCl_2_ to the medium to measure Ca^2+^ entry. In this setting, TRIC-A expression promoted Ca^2+^ release from the ER (Fig 2B). Importantly, it induced a significant reduction in the rate of Ca^2+^ influx, the peak amplitude, and sustained [Ca^2+^]_i_ elevation by 40%, 47%, and 41%, respectively (Fig 2C and 2D). These effects of TRIC-A expression on SOCE were similar when ER Ca^2+^ was depleted with BHQ alone (without caffeine) in these cells (S3A Fig). The influx rate and amplitudes of peak and sustained cytosolic Ca^2+^ elevation were also significantly reduced by 25%, 20%, and 16%, respectively (S3B and S3C Fig). Peak cytosolic Ca^2+^ levels in response to BHQ-mediated store depletion were slightly higher in TRIC-A–expressing cells, although the difference was not statistically significant (S3D Fig). Importantly, when overexpressed in rat basophilic leukemia cell line (RBL-2H3), which expresses a large, endogenous STIM1/Orai1-mediated SOCE (S3E Fig), TRIC-A significantly reduced the rate of Ca^2+^ influx as a measure of SOCE in classical Ca^2+^ readdition experiments by 44% (S3F Fig), though steady-state amplitudes of cytosolic Ca^2+^ elevation remained barely affected (S3G Fig). Moreover, knockdown of endogenous TRIC-A in mouse atrial muscle cell line (HL-1) was tested for its impact on SOCE. In line with previous finding [28], HL-1 cardiomyocytes exhibited SOCE, following store depletion with caffeine + BHQ (S4B Fig). Knockdown of *TRIC-A* by small interfering RNA (siRNA) to 53% significantly enhanced endogenous SOCE as compared to controls conducted using a scrambled siRNA (si-scr) (S4A Fig). TRIC-A knockdown significantly accelerated the initial rate of Ca^2+^ influx in Ca^2+^ readdition experiments by 31% (S4C Fig) and enhanced peak and sustained cytosolic Ca^2+^ rises (S4D Fig). These findings suggest that TRIC-A attenuates the SOCE pathway via a mechanism that is unrelated to ER Ca^2+^ recycling.

These effects of TRIC-A expression on SOCE were further tested by examining agonist-stimulated Ca^2+^ signals in wild-type HEK293 cells that lack RyR2 expression (Fig 2E and S5A Fig). Peak increases in [Ca^2+^]_i_ in response to stimulation with carbachol (CCh) + BHQ (Fig 2F) or CCh alone (S5B Fig) were not significantly altered by TRIC-A expression. However, as seen in Fig 2A, both peak and sustained [Ca^2+^]_i_ elevations, as well as the rate of Ca^2+^ entry, were significantly inhibited by, respectively, 39%, 33%, and 35% (Fig 2G and 2H). Importantly, SOCE attenuation in TRIC-A–expressing cells was not due to diminished expression of its 2 major molecular components, STIM1 and Orai1, as confirmed by immunoblotting (S6A and S6B Fig).

In an attempt to elucidate the impact of TRIC-A expression on agonist-dependent Ca^2+^ signaling responses, sustained via SOCE, we investigated [Ca^2+^]_i_ responses in HEK293 cells at various [CCh]. As reported previously [29], CCh-induced [Ca^2+^]_i_ responses varied with an increase in stimulus intensities, with baseline Ca^2+^ oscillations at low [CCh] and sustained elevations at maximal [CCh]. We observed TRIC-A–induced variation in the Ca^2+^ signaling pattern at each [CCh]. We categorized these responses into 4 groups between 6- and 10-min time frames: no sustained response to CCh or exclusively ER Ca^2+^ mobilization from stores (no response), oscillations that return to basal levels after each release event (baseline oscillations) (Fig 2I), oscillations that returned to an elevated plateau above basal level (oscillations + elevated plateau) (Fig 2J), and sustained elevation above basal level without oscillations (elevated plateau) (Fig 2K). When stimulated with 5 μM CCh (Fig 2L), 57% of control cells did not display a response, and the distribution of responses with baseline oscillations, oscillations + elevated plateau, and elevated plateau were 29%, 4%, and 10%, respectively. TRIC-A expression shifted the distribution pattern with 5 μM CCh to the lower response categories, with 69% nonresponders and no cells displaying an elevated plateau of [Ca^2+^]_i_. Upon stimulation with 100 μM CCh (Fig 2M), the majority of control cells (56%) displayed an elevated plateau, and only 16% showed baseline oscillations. TRIC-A expression reduced the fraction of cells showing elevated plateau responses to 31% while increasing the proportion cells with baseline oscillations to 27%. Sustained elevation or oscillations in agonist-stimulated HEK293 cells are driven by SOCE [29,30]. Thus, the TRIC-A–induced changes are likely based on the reduction in SOCE.

### TRIC-A delays cyclic Ca^2+^ refilling upon store depletion

**Fig 3:**
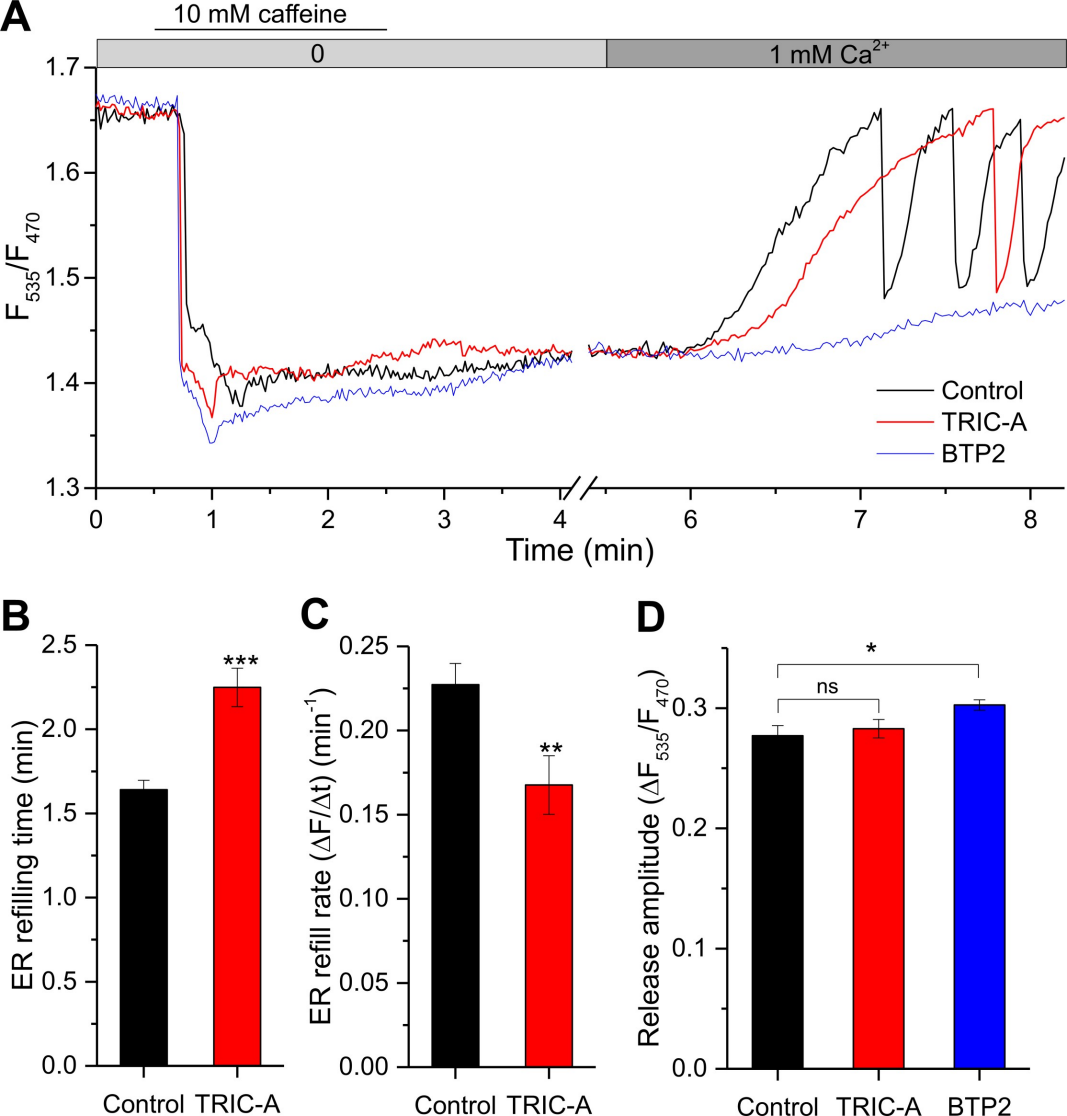
TRIC-A delays cyclic Ca^2+^ refilling upon store depletion. (A) [Ca^2+^]_ER_-sensitive D1ER traces representing ER Ca^2+^ depletion with 10 mM caffeine, followed by ER refilling upon 1 mM [Ca^2+^]_o_ addition in an mCherry-ER-3– (control, black) or TRIC-A-mCherry (TRIC-A, red)–transfected HEK293_RyR2 cell or diminished refilling in a 3 μM BTP2-incubated control cell (BTP2, blue). Bar graphs show mean ± SEM values for (B) ER refilling time and (C) ER refill rate in TRIC-A (+) cells (*n* = 45) versus controls (*n* = 51), and (D) ER Ca^2+^ release peak amplitude in TRIC-A (+) cells (*n* = 45) and BTP2-incubated cells (*n* = 36) versus controls (*n* = 51); *p < 0.05, **p < 0.01, ***p < 0.001. Underlying data in panels (A–D) are included in S1 Data. BTP2, N-[4-[3,5-Bis(trifluoromethyl)pyrazol-1-yl]phenyl]-4-methylthiadiazole-5-carboxamide; D1ER, genetically encoded ER-targeted Ca^2+^ sensor; ER, endoplasmic reticulum; HEK293, human embryonic kidney 293; ns, nonsignificant; RyR, ryanodine receptor; TRIC, trimeric intracellular cation.

SOCE modulation by TRIC-A was further confirmed in experiments monitoring the time course of [Ca^2+^]_ER_ during store depletion by caffeine and its subsequent refilling by elevation of [Ca^2+^]_o_ from nominally free to 1 mM in HEK293_RyR2 cells (Fig 3A). Similar to the spontaneous oscillations observed earlier (S1 Fig), the time course of ER Ca^2+^ store refilling after a caffeine-induced ER discharge was significantly slowed, and luminal Ca^2+^ oscillations induced by [Ca^2+^]_o_ were delayed in onset and had reduced frequency. TRIC-A expression prolonged the time required to complete ER refilling by 0.61 ± 0.06 min (Fig 3B), reducing the rate of refilling by 26% compared to controls (Fig 3C). Likewise, in the presence of 3 μM BTP2, ER refilling was blocked following store depletion by caffeine (Fig 3A) because of the elimination of SOCE, while RyR2-mediated Ca^2+^ mobilization was even moderately increased (Fig 3D). Hence TRIC-A impacts both ER Ca^2+^ store refilling and generation of cytosolic Ca^2+^ oscillations. Notably, TRIC-A did not enhance ER Ca^2+^ release induced by caffeine (Fig 3D). In aggregate, these findings strongly suggest TRIC-A as a potential regulator of SOCE during oscillatory Ca^2+^ signaling.

### TRIC-A inhibits Orai1-mediated Ca^2+^ release-activated Ca^2+^ current (*I*_CRAC_) and STIM1–Orai1 interaction upon store depletion

**Fig 4:**
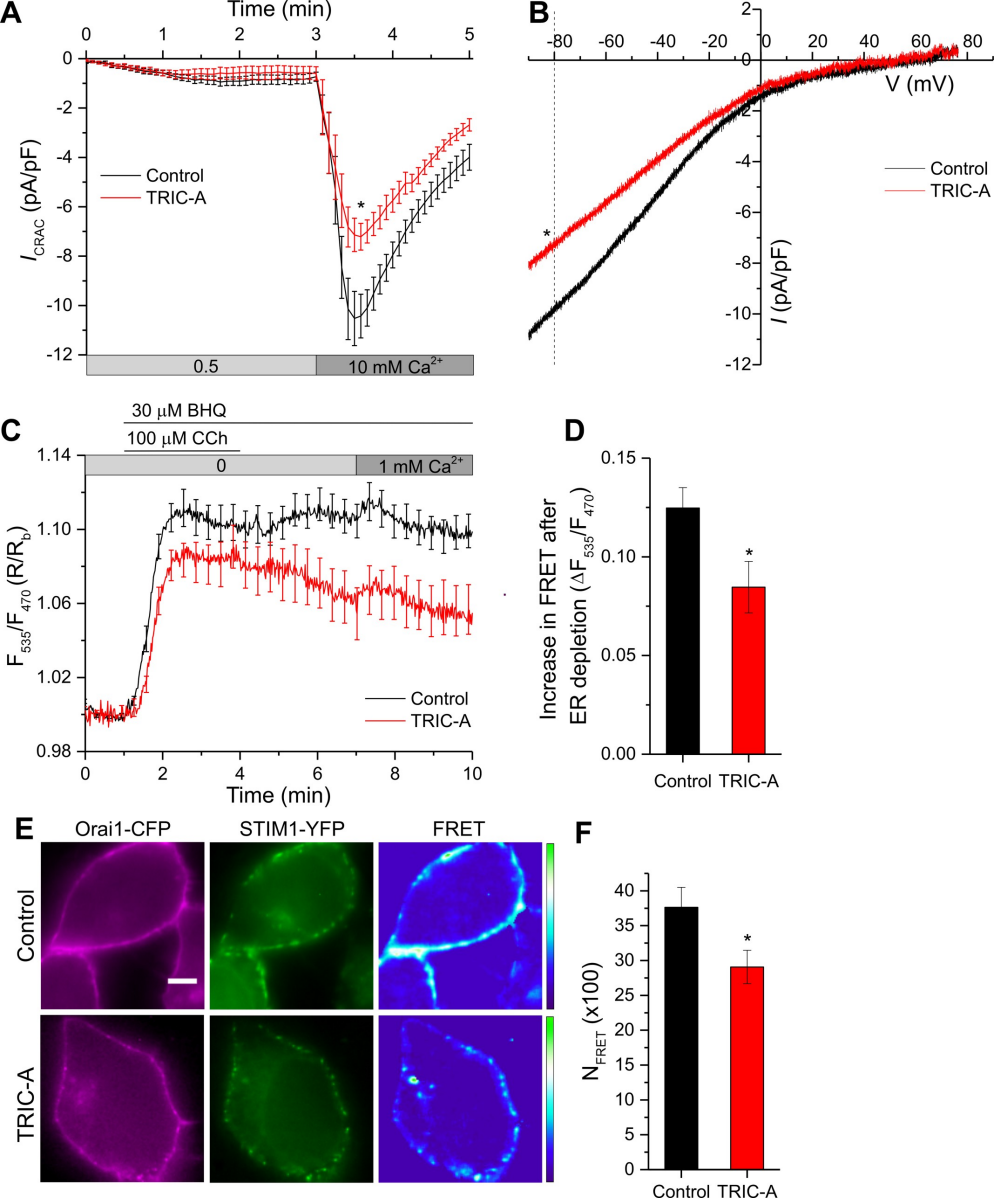
**TRIC-A inhibits Orai1-mediated *I***_**CRAC**_ **(A, B) and STIM1–Orai1 interaction (C–F) upon store depletion.** (A) Whole-cell voltage-clamp experiments show time course of *I*_CRAC_ at −80 mV mediated by YFP-STIM1 + Orai1-CFP, coexpressed along with mCherry-ER-3 (control, *n* = 13) or TRIC-A-mCherry (TRIC-A, *n* = 11) in HEK293 cells. Current activation was mediated by ER store depletion with 10 mM EGTA in the patch pipette solution, and stepwise current increment was recorded at 0.5 and 10 mM Ca^2+^ in the bath solution. (B) Traces show representative peak I–V relationship in control and TRIC-A groups at 10 mM Ca^2+^. (C) Traces show dynamic FRET between STIM1-CFP and YFP-Orai1, co-transfected in HEK293 cells along with mCherry-ER-3 (control, *n* = 20) or TRIC-A-mCherry (TRIC-A, *n* = 13). (D) Increase in STIM1-Orai1 FRET shown in (C) upon ER depletion with 100 μM CCh + 30 μM BHQ in TRIC-A cells versus controls. (E) Representative epifluorescence images of ER-depleted (100 μM CCh + 30 μM BHQ) HEK293 cell expressing Orai1-CFP (left, magenta) + STIM1-YFP (middle, green) and corresponding FRET (right) in absence (Control, top) and presence (TRIC-A, bottom) of coexpressed TRIC-A. Scale bar = 5 μm. (F) Bars show N_FRET_ (× 100) in TRIC-A-transfected cells (*n* = 15) versus controls (*n* = 16). *p < 0.05, mean values ± SEM are shown. Underlying data in panels (A–D) and (F) are included in S1 Data. BHQ, 2,5-Di-*t*-butyl-1,4-benzohydroquinone; CCh, carbachol; CFP, cyan fluorescent protein; ER, endoplasmic reticulum; FRET, Förster resonance energy transfer; HEK293, human embryonic kidney 293; *I*_CRAC_, Ca^2+^ release-activated Ca^2+^ current; I–V, current–voltage; N_FRET_, normalized FRET; Orai1, Ca^2+^-release–activated Ca^2+^ channel 1; STIM1, stromal interaction molecule 1; TRIC, trimeric intracellular cation; YFP, yellow fluorescent protein.

To more directly examine the impact of TRIC-A on SOCE, we measured the SOCE-associated, inwardly rectifying, Ca^2+^ release-activated Ca^2+^ currents (*I*_CRAC_). Yellow fluorescent protein (YFP)-STIM1 and Orai1-CFP were coexpressed along with mCherry-ER-3 (control) or TRIC-A-mCherry (TRIC-A), and *I*_CRAC_ was measured using a whole-cell patch-clamp technique (Fig 4A). Fig 4B illustrates a representative current–voltage (I–V) relationship of the currents. While there was no change in reversal potential, the magnitude of the inward current was significantly reduced (current density at −80 mV was −8.30 ± 0.87 pA/pF in TRIC-A-expressing cells versus −11.82 ± 0.99 pA/pF in controls).

Because gating of the Orai1 channel is triggered by the physical interaction of the channel with STIM1, we used the Förster resonance energy transfer (FRET) technique to assess the dynamic assembly of the STIM1-CFP/YFP-Orai1 complex in response to ER Ca^2+^ store depletion (Fig 4C). Stimulation of cells with BHQ + CCh induced a fast and distinct increase of FRET in control and TRIC-A–expressing cells. Peak FRET increase was significantly diminished in cells expressing TRIC-A (by 32%) as compared to controls (Fig 4D). Interestingly, while a high FRET was maintained in control cells, FRET measured in TRIC-A–expressing cells declined markedly to about 50% within 10 min, despite the continued presence of BHQ. This finding suggested that TRIC-A profoundly affects the assembly of the STIM1/Orai1 complex at ER–PM junctions by directly interfering with STIM1–Orai1 interaction. The decline in FRET suggests that STIM1/Orai1 interaction in TRIC-A–expressing cells might be less stable compared to that in control cells. Additional evaluation of steady-state interactions between STIM1 and Orai1 fusion proteins by normalized FRET (N_FRET_) [31] in store-depleted cells confirmed a substantial interference of TRIC-A with the coupling process within this Ca^2+^ entry channel complex (Fig 4E and 4F).

### TRIC-A interacts with STIM1 and coclusters at ER–PM junctions upon store depletion

**Fig 5:**
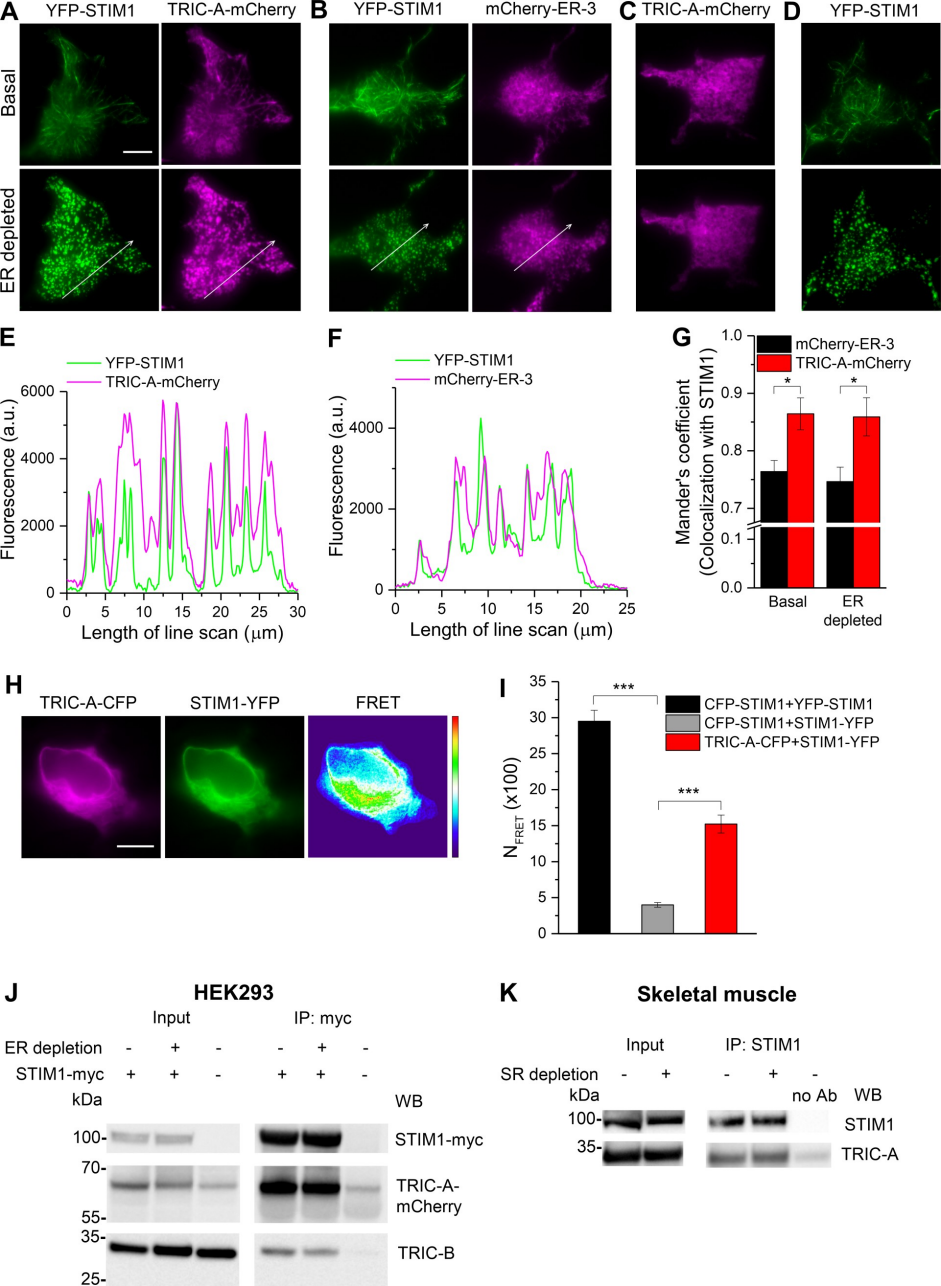
TRIC-A interacts with STIM1 and coclusters at ER–PM junctions upon store depletion. Representative TIRF images of basal (top) and ER-depleted (100 μM CCh + 30 μM BHQ) (bottom) HEK293 cell expressing (A) YFP-STIM1 (left, green) and TRIC-A-mCherry (right, magenta), (B) YFP-STIM1 (left, green) and mCherry-ER-3 (right, magenta), and (C) TRIC-A-mCherry, and (D) YFP-STIM1. Scale bar = 10 μm. (E, F) Line scans of proteins in ER-depleted cell shown in (A, B). (G) Mean ± SEM bars show Mander’s coefficient for proportion of mCherry-ER-3 (*n* = 9) and TRIC-A-mCherry (*n* = 8) colocalized with STIM1 under basal and ER-depleted conditions, *p < 0.05. (H) Representative epifluorescence images of HEK293 cell coexpressing TRIC-A-CFP (left, magenta) and STIM1-YFP (middle, green) and corresponding FRET (right). Scale bar = 10 μm. (I) Bars show mean ± SEM values for N_FRET_ (× 100) of TRIC-A-CFP + STIM1-YFP (*n* = 30) compared to CFP-STIM1 + YFP-STIM1 (positive control, *n* = 10) and CFP-STIM1 + STIM1-YFP (negative control, *n* = 12), ***p <0.001. (J, K) Representative Co-IP of (J) TRIC-A-mCherry and TRIC-B with STIM1-myc in HEK293 cells and (K) TRIC-A with STIM1 in murine skeletal muscles. Lysates were obtained from (J) basal (−) and ER-depleted (+) (100 μM CCh + 30 μM BHQ) HEK293 cells and (K) basal (−) and SR-depleted (+) (30 mM caffeine + 30 μM BHQ) murine skeletal muscles, *n* = 3 independent experiments. Underlying data in panels (E–G) and (I) are included in S1 Data. a.u., arbitrary unit; BHQ, 2,5-Di-*t*-butyl-1,4-benzohydroquinone; CCh, carbachol; CFP, cyan fluorescent protein; Co-IP, coimmunoprecipitation; ER, endoplasmic reticulum; FRET, Förster resonance energy transfer; HEK293, human embryonic kidney 293; N_FRET_, normalized FRET; PM, plasma membrane; SR, sarcoplasmic reticulum; STIM1, stromal interaction molecule 1; TIRF, total internal reflection fluorescence; TRIC, trimeric intracellular cation; WB, western blot; YFP, yellow fluorescent protein.

Next, we characterized the impact of TRIC-A on the initial molecular processes of SOCE activation initiated by the dissociation of luminal Ca^2+^ from the EF-hand motifs in STIM1, leading to its oligomerization and translocation into discrete punctae within ER–PM junctions [14–16]. As observed by total internal reflection fluorescence (TIRF) microscopy, the formation of YFP-STIM1 punctae in HEK293 cells was comparable to that in TRIC-A–expressing cells, indicating that the initial process of STIM1 activation was unaffected by TRIC-A. Both YFP-STIM1 and TRIC-A-mCherry, when coexpressed, localized in ER membranes forming network-like structures at basal, unstimulated conditions (Fig 5A, top), comparable to coexpressed YFP-STIM1 and mCherry-ER-3 (ER marker) (Fig 5B, top). Interestingly, TRIC-A-mCherry dynamically translocated to cocluster with YFP-STIM1 within punctae upon ER Ca^2+^ store depletion (Fig 5A, bottom), as evident from the line-scan analysis (Fig 5E; see also S1 Video). Similarly, mCherry-ER-3 redistributed with clustered YFP-STIM1 upon ER Ca^2+^ store depletion (Fig 5B, bottom), illustrated by the line scan in Fig 5F. Moreover, calculation of Mander’s coefficient suggested a significantly higher extent of YFP-STIM1 colocalization with TRIC-A-mCherry than with mCherry-ER-3 under both basal and ER-depleted conditions (Fig 5G). Notably, clustering of TRIC-A-mCherry required overexpressed STIM1 because clustering was absent when TRIC-A was expressed alone (Fig 5C, also see S2 Video). This might be interpreted in terms of the requirement for a distinct stoichiometry of the interaction that is enabled only at certain STIM1 levels. In contrast, YFP-STIM1 did not require coexpression of TRIC-A to form punctae upon ER Ca^2+^ store depletion (Fig 5D). Besides, a constitutive physical interaction between STIM1-YFP and TRIC-A-CFP was suggested by determination of N_FRET_ values from epifluorescence images (Fig 5H and 5I), which was significantly higher than negative control, CFP-STIM1, and STIM1-YFP.

Coimmunoprecipitation (Co-IP) experiments confirmed the physical interaction between TRIC-A and STIM1 in both HEK293 cells and in native skeletal muscle. When reconstituted in HEK293 cells (Fig 5J), TRIC-A-mCherry immunoprecipitated with STIM1-myc even at basal, unstimulated conditions. In control IPs (absence of STIM1-myc expression), TRIC-A-mCherry immunoreactivity was only faint (0.18 ± 0.03-fold intensity versus bands with STIM1-myc). Of note, STIM1 complexes were found to contain also some endogenous TRIC-B. Similarly, in reciprocal Co-IP, endogenous STIM1 immunoprecipitated with myc-TRIC-A (S7 Fig). Interestingly, no increase in TRIC-A-STIM1 association was observed upon store depletion, suggesting that TRIC-A-STIM1 interaction was independent of the Ca^2+^ filling state of the ER stores (Fig 5J). This was further confirmed by the coexpression of TRIC-A-mCherry with the EF-hand mutant of STIM1 (YFP-STIM1-D76A). This STIM1 mutant is constitutively active, thereby forming preclusters under basal, unstimulated conditions, and displays no significant increase in punctae aggregation following store depletion [32]. TRIC-A coclustered with YFP-STIM1-D76A independently of store depletion when coexpressed in the same cell (S8A Fig), as evident from line-scan analysis (S8B Fig) and Mander’s coefficient (S8C Fig). Thus, TRIC-A-STIM1 interaction appears to be independent of ER Ca^2+^ store depletion. YFP-STIM1-D76A expression generated constitutive Ca^2+^ influx, leading to sustained cytosolic Ca^2+^ rises in the absence of oscillatory Ca^2+^ cycling. Ca^2+^ oscillations were never observed in HEK293 cells lacking overexpression of RyR2 (S8D Fig).

STIM1–TRIC-A interaction was further confirmed using native skeletal muscle isolated from hind limbs of mouse where endogenous TRIC-A coimmunoprecipated with STIM1 (Fig 5K). Considering a physical interaction between TRIC-A and STIM1 that interferes and modulates spatiotemporal Ca^2+^ signaling via STIM1/Orai1, we next focused on a quantitative analysis of the underlying productive translocation and clustering processes.

### TRIC-A affects kinetics and extent of STIM1-Orai1 puncta formation

**Fig 6:**
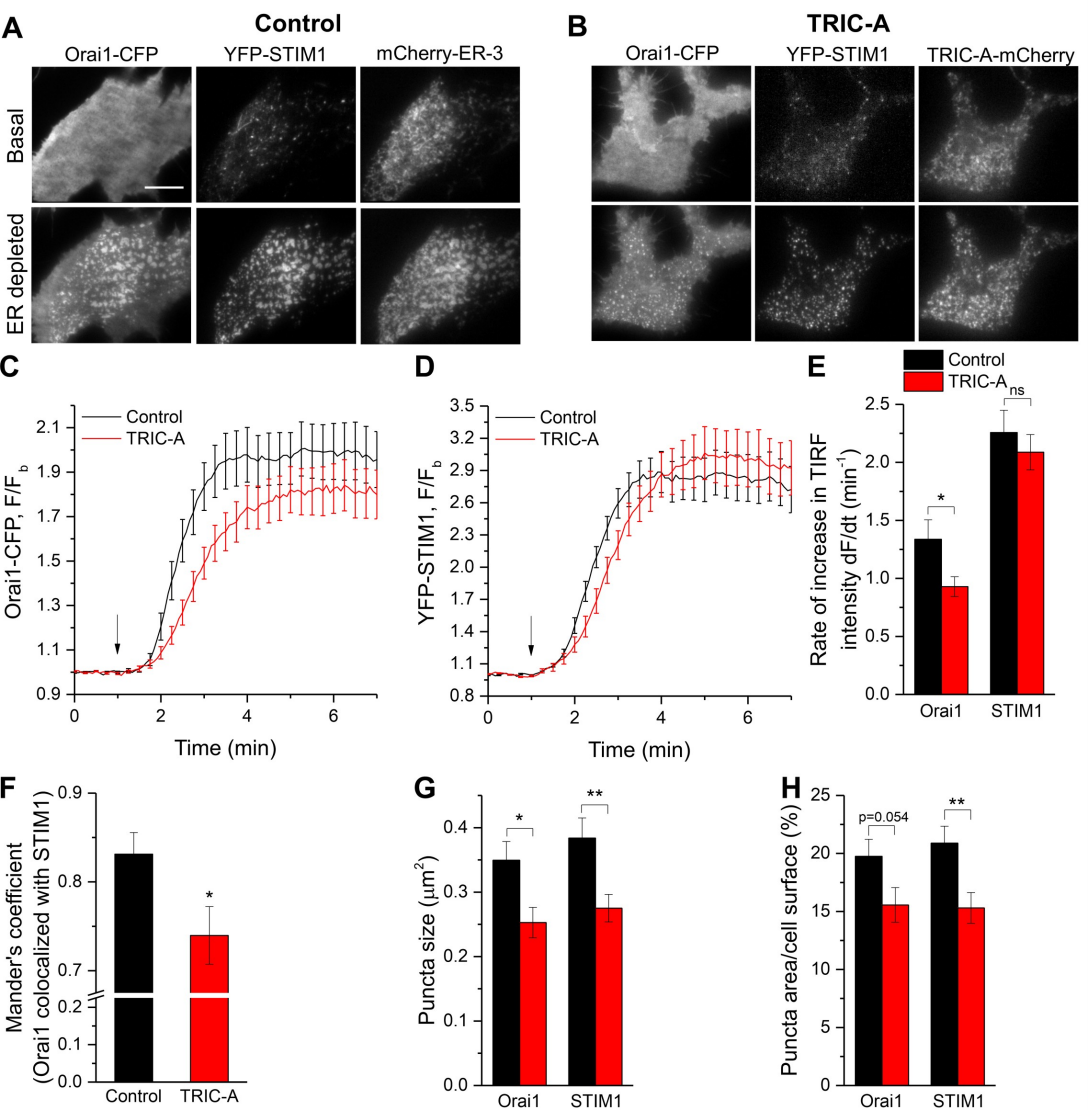
TRIC-A affects kinetics and extent of STIM1-Orai1 puncta formation upon store depletion. (A, B) TIRF images of basal (top) and ER-depleted (100 μM CCh + 30 μM BHQ) (bottom) cell expressing Orai1-CFP (left, Orai1) and YFP-STIM1 (middle, STIM1) along with mCherry-ER-3 (right) (A, control) or TRIC-A-mCherry (right) (B, TRIC-A). Scale bar = 10 μm. (C, D) Traces show kinetics of Orai1-CFP and YFP-STIM1 TIRF intensity upon ER depletion (arrow) in TRIC-A (+) cells versus controls (*n* = 18 each). (E) Rate of increase in Orai1 and STIM1 TIRF intensity upon ER depletion shown in (C, D). (F) Mander’s coefficient showing proportion of Orai1 colocalized with STIM1, (G) average puncta size (μm^2^), and (H) puncta area relative to cell surface area (%) of Orai1 and STIM1 in TRIC-A (+) cells versus controls. *p < 0.05, **p < 0.01; mean values ± SEM are shown. Underlying data in panels (C–H) are included in S1 Data. BHQ, 2,5-Di-*t*-butyl-1,4-benzohydroquinone; CCh, carbachol; CFP, cyan fluorescent protein; ER, endoplasmic reticulum; Orai1, Ca^2+^-release–activated Ca^2+^ channel 1; STIM1, stromal interaction molecule 1; TIRF, total internal reflection fluorescence; TRIC, trimeric intracellular cation; YFP, yellow fluorescent protein.

SOCE depends on the assembly of the STIM1/Orai1 complex within ER–PM junctions. Any impairment of the clustering of these proteins may hinder their interaction and suppress Orai1 channel activation and SOCE [18–20]. Consequently, we analyzed the impact of TRIC-A on the clustering of STIM1 with Orai1 within ER–PM junctions. In HEK293 cells expressing both Orai1-CFP and YFP-STIM1, together with mCherry-ER-3 (Fig 6A, control) or TRIC-A-mCherry (Fig 6B, TRIC-A), the proteins clustered and formed discrete punctae upon ER Ca^2+^ store depletion. The rate of Orai1 puncta formation was significantly delayed in cells expressing TRIC-A (Fig 6C and 6E). TRIC-A slightly delayed STIM1 puncta formation, though statistically insignificant (Fig 6D and 6E).

Importantly, TRIC-A significantly reduced the colocalization of STIM1 and Orai1 within each puncta (Fig 6F). The overall size distribution of punctae for Orai1 (S9A Fig) and STIM1 (S9B Fig) was significantly altered by TRIC-A expression as compared to controls (***p < 0.001; χ^2^ test). TRIC-A cells displayed a higher fraction of punctae displaying the smallest size (0.04–0.2 μm) but reduced numbers of large clusters (0.4–1.4 μm) when compared to controls. Hence, average puncta size for both Orai1 and STIM1 was significantly reduced in TRIC-A–expressing cells (Fig 6G), indicating a reduced footprint for the STIM1/Orai1 puncta in the cell (Fig 6H). However, TRIC-A expression did not alter the number of punctae per membrane area (density) for both Orai1 and STIM1 (S9C Fig).

Notably, these effects on Orai1 and STIM1 puncta formation and interaction were not due to differences in protein expression levels because the basal epifluorescence intensity of both proteins were unaffected by TRIC-A expression (S6C Fig). Together, the data in Figs 5 and 6 indicate that TRIC-A interacts with STIM1 and is recruited by STIM1 to ER–PM junctions. Within the junctions, TRIC-A impedes clustering of STIM1 with Orai1 as well as their interaction.

## Discussion

**Fig 7:**
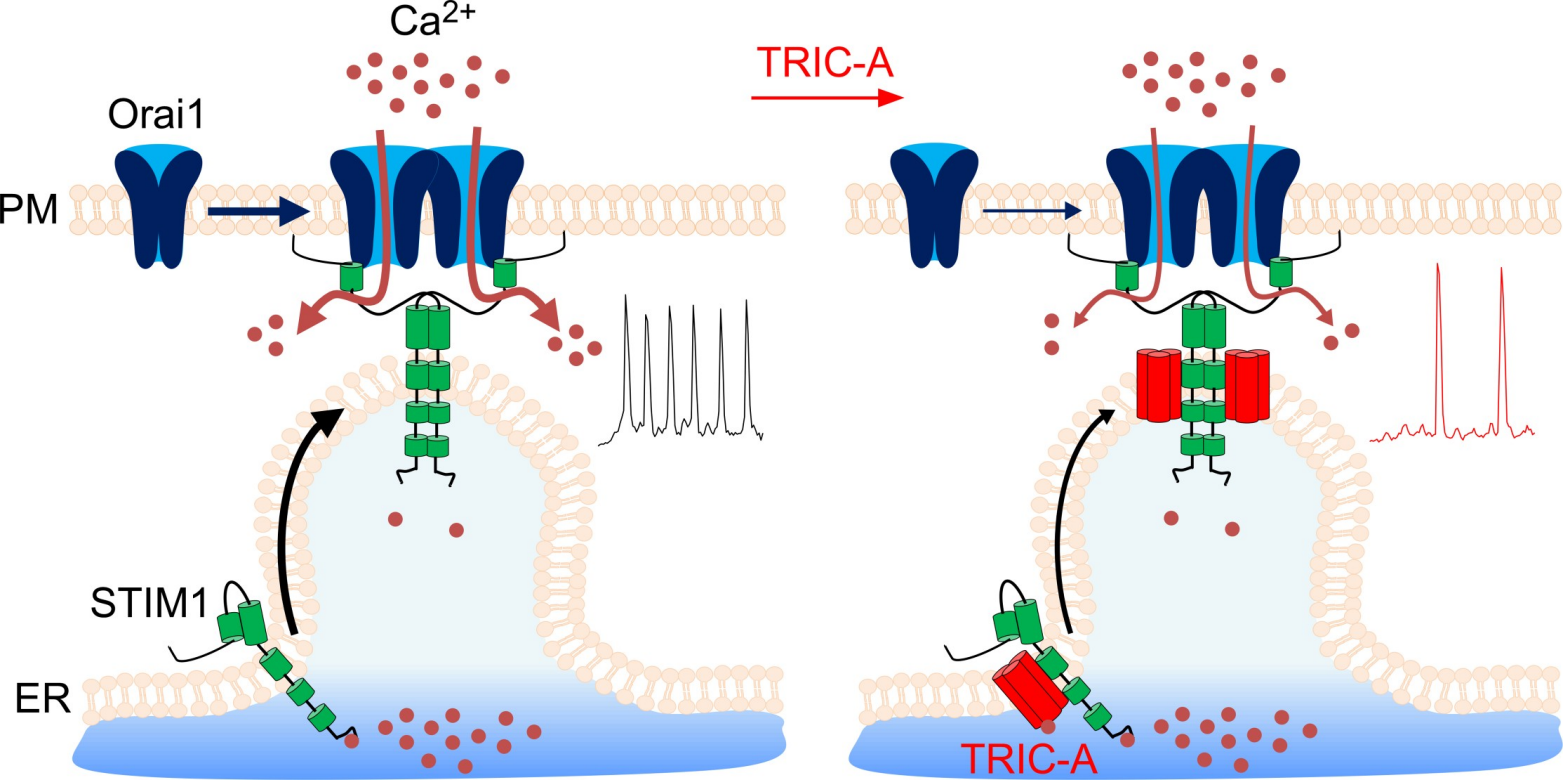
Schematic diagram illustrating the role of TRIC-A in limiting SOCE and oscillatory Ca^2+^ signals by interfering with STIM1/Orai1 complex assembly. Left: Upon ER Ca^2+^ depletion via RyR/IP_3_R, STIM1 loses bound Ca^2+^ from its EF-hand, undergoes a conformational change, oligomerizes, and translocates into clusters at ER–PM junctions. STIM1 then recruits Orai1 channels into the clusters and activates the Ca^2+^-selective pore for subsequent Ca^2+^ entry into the cells to sustain RyR/IP_3_R-triggered Ca^2+^ oscillations. Right: TRIC-A translocates along with STIM1 to ER–PM junctions, where it interferes with STIM1/Orai1 coupling, which consequently limits the Ca^2+^ influx via Orai1 (SOCE) and the frequency of cytosolic Ca^2+^ oscillations. ER, endoplasmic reticulum; *I*_CRAC_, Ca^2+^ release-activated Ca^2+^ current; IP_3_R, inositol 1,4,5-triphosphate receptor; Orai1, Ca^2+^-release–activated Ca^2+^ channel 1; PM, plasma membrane; RyR, ryanodine receptor; SOCE, store-operated Ca^2+^ entry; STIM1, stromal interaction molecule 1; TRIC, trimeric intracellular cation.

The data presented above demonstrate a novel, to our knowledge, function of TRIC-A in SOCE signaling. As summarized in Fig 7, we present multiple lines of evidence to demonstrate that TRIC-A channels interfere with the STIM1/Orai1 assembly and function: (i) TRIC-A is recruited by STIM1 to form punctae within the ER–PM junctions following Ca^2+^ store depletion, (ii) TRIC-A colocalizes with STIM1 and Orai1 punctae, (iii) TRIC-A interacts with STIM1, (iv) TRIC-A decreases STIM1/Orai1 interaction, and (v) TRIC-A attenuates *I*_CRAC_ and SOCE. Together, our findings suggest that TRIC-A is a negative regulator of SOCE that shapes cytosolic Ca^2+^ oscillations by modulating STIM1/Orai1 assembly and ER Ca^2+^ recycling.

To our knowledge, this is the first report of TRIC-A interference with STIM1/Orai1 assembly and function. Our data further reveal that the recruitment of TRIC-A into ER–PM junctions is triggered by ER Ca^2+^ depletion but is mediated by its interaction with STIM1 rather than its own ER Ca^2+^-sensing function [4]. STIM1-D76A, a STIM1 mutant with disrupted luminal Ca^2+^ binding in its N-terminal EF-hand domain, displays constitutive clustering at ER–PM junctions [32]. Preclustering of this STIM1 mutant is sufficient for promoting coclustering of TRIC-A in ER–PM junctions in the absence of store depletion. Of note, endogenous STIM1 per se appeared insufficient to produce significant clustering of overexpressed TRIC-A, implying that TRIC-A stoichiometry relative to STIM1 is critical for the extent of cotargeting into STIM1 clusters. In cells coexpressing Orai1 and STIM1, TRIC-A attenuates the assembly of STIM1 and Orai1, as evidenced by the reduction in the FRET signal that is generated because of an interaction between the 2 proteins. The decreased FRET, together with the changes in the pattern of clustering of Orai1/STIM1, can account for the decrease in SOCE. Importantly, the refilling of ER Ca^2+^ stores is attenuated by TRIC-A. Our findings provide strong evidence that this delay is due to disruption of STIM1/Orai1 assembly and consequent decrease in SOCE, which is required for refilling the intracellular Ca^2+^ stores. An additional, more direct effect of TRIC-A on the SERCA pump cannot be ruled out.

TRIC-A has been previously shown to modulate Ca^2+^ handling in muscle cells by affecting RyR function [1,5,6]. The present study demonstrates that RyR2-mediated spontaneous Ca^2+^ oscillations are dependent on SOCE. Thus, by limiting SOCE, TRIC-A modulates SR/ER refilling and the frequency of the oscillations. In addition, the enhanced Ca^2+^ discharge amplitude during each oscillation is in line with the reported monovalent conductance generated by TRIC channels across the ER membrane, which has been proposed to facilitate Ca^2+^ efflux from the ER via K^+^ counterflux [1,2]. Instead, when both TRIC isoforms were knocked out, embryonic cardiomyocytes were reported to display diminished RyR2-triggered spontaneous Ca^2+^ oscillations because of compromised K^+^ counterflux [1]. Of note, the peak amplitude of RyR2-mediated oscillatory Ca^2+^ cycling was indeed enhanced by TRIC-A expression, while this phenomenon was not observed for single caffeine-mediated depletion of the ER in the absence of cellular Ca^2+^ cycling. This finding might be taken as an indication that facilitation of ER Ca^2+^ mobilization by TRIC-A is operative only in the setting of oscillatory Ca^2+^ signaling, potentially requiring distinct local changes in Ca^2+^ at the TRIC channel complex.

Our study provides evidence for modulatory interaction of TRIC-A with the SOCE mechanism of Ca^2+^ entry as the basis of its impact on Ca^2+^ oscillation pattern. This conclusion has been obtained by both heterologous reconstitution of signaling in HEK293 cells and corroborated in muscle tissue endogenously expressing TRIC-A and the STIM1/Orai1 SOCE machinery.

While the precise role of SOCE in muscle physiology is not fully understood, SOCE does play an important role in skeletal muscle physiology ranging development, differentiation, contractile function, and resistance against fatigue [33,34]. Skeletal muscle hypotonia and weakness are characteristics of STIM1- and Orai1-deficient patients and mice [34,35]. While physiological SOCE is required for refilling SR Ca^2+^ stores and proper muscle development and function, excessive SOCE seems to be detrimental, leading to muscular dystrophy [33,34]. Increased STIM1 function has also been associated with cardiac hypertrophy and hypertension, characterized by enhanced spontaneous Ca^2+^ transients and downstream transcription coupling, and decrease in its function has been proposed to ameliorate these cardiac disorders [36–38]. Thus, tight regulation of STIM1 function and SOCE is critical for maintaining proper muscle physiology based on oscillatory Ca^2+^ signals. Overloading of SR Ca^2+^ stores, aberrant ER-mitochondrial connections, and mitochondrial dysfunction associated with TRIC-A–deficient muscle cells are indeed reminiscent of effects caused by increased STIM1 function. Thus, increased SOCE, in the absence of TRIC-A, as we have observed with TRIC-A knockdown in the cardiac muscle cell line HL-1, can cause ER and mitochondrial Ca^2+^ overload. It is also of interest that another ER-resident protein, SOCE-associated regulatory factor (SARAF), when overexpressed, can mitigate the effect of STIM1 in cardiac hypertrophy and diastolic dysfunction [39]. Interestingly, SARAF, like TRIC-A, is recruited to ER–PM junctions by STIM1, where it increases Ca^2+^-dependent inactivation of Orai1 [40]. Thus, 2 ER proteins negatively modulate STIM1/Orai1 function in muscle cells to limit SOCE and prevent SR Ca^2+^ overload. Further studies will be required to evaluate their individual effects on SOCE and the consequent physiological impact in muscles.

In summary, our findings suggest that TRIC-A–mediated regulation of SOCE can play a major role in regulating Ca^2+^ homeostasis in muscle cells. In addition, loss of TRIC-A, and a consequent gain of SOCE can disrupt Ca^2+^ handling, resulting in long-term consequences and dysfunction. Indeed, TRIC-A variants have been associated with hypertension in patients [5]. Since both TRIC-A and SOCE impact skeletal, cardiac, and smooth muscle function, the TRIC-A–SOCE interaction might reflect a common mechanism to prevent SR Ca^2+^ overload and dysfunction in these different types of muscles. Therapies targeting SOCE could be beneficial in patients with TRIC-A defects.

## Materials and methods

### Ethical statement

The experimental procedure was approved by the ethics commission of the Federal Ministry of Science, Research and Economics of the Republic of Austria (BMWF-66.010/0101-WF/V/3b/2016). The experiments were carried out in accordance with the Directive of the European Parliament and of the Council of 22 September 2010 (2010/63/EU). C57BL/6 mice (12 to 16 weeks, male) were anesthetized with ketamine (100 mg/kg, i.p.) and xylazine (10 mg/kg, i.p.) and killed via cervical dislocation. As described previously, the skeletal muscles were dissected from hind limbs of mice [41].

### Reagents and constructs

All reagents used were of molecular biology grade, purchased from Sigma-Aldrich (St. Louis, MO, USA) unless specified otherwise. HEK293 cells stably expressing human RyR subtype 2 (HEK293_RyR2) and D1ER constructs were provided by Roland Malli (Medical University of Graz, Graz, Austria). We cloned TRIC-A fusion constructs in pCMV-Myc, pECFP-N1, and pEYFP-N1 vectors (Clontech, Saint-Germain-en-Laye, France) from untagged mouse TRIC-A, provided by Hiroshi Takeshima (Kyoto University, Kyoto, Japan). YFP-STIM1, Orai1-CFP, STIM1-myc, mCerulean-ER-5, and mCherry-ER-3 were obtained from Indu Suresh Ambudkar (NIH, Bethesda, MD, USA). STIM1-CFP, STIM1-YFP, CFP-STIM1, YFP-Orai1, YFP-Orai1-E106Q, and YFP-STIM1-D76A were provided by Christoph Romanin (University of Linz, Linz, Austria). CFP-GPI was provided by Wolfgang Schreibmayer (Medical University of Graz, Graz, Austria).

### Cell culture and transfection

HEK293, HEK293_RyR2, and RBL-2H3 cells were cultured in DMEM supplemented with 10% FBS (Gibco 10270; Thermo Fisher Scientific, Bohemia, NY, USA), and 10 mM HEPES. HL-1 cells were cultured in fibronectin (0.5% w/v)/gelatin (0.02% w/v)-coated dishes and maintained in Claycomb medium containing 10% FBS, 0.1 mM norepinephrine, and 2 mM L-glutamine. All cell lines were maintained in an incubator at 37°C, 5% CO_2_ in 100 IU/ml penicillin and 100 μg/ml streptomycin, except in 250 μg/ml geneticin and 5 μg/ml puromycin for HEK293_RyR2 cells. The cells at 50%–60% confluence in 35-mm dishes were transiently transfected for 6 h with required constructs, as indicated in the text, using FugeneHD (Promega, Walldorf, Germany) or Lipofectamine 2000 (Invitrogen, Vienna, Austria) as per manufacturer’s instructions. Control cells were mock transfected with an ER marker, mCherry-ER-3 or YFP plasmid, as indicated in the text. For siRNA transfection, 80 nmol siRNA against mTRIC-A (sc-154461; Santa Cruz Biotechnology, Dallas, TX, USA) or scrambled control (1027280, Qiagen, Hilden, Germany) were used, following manufacturer’s instructions. Experiments were performed 40–48 h after transfection.

### [Ca^2+^]_i_ imaging and FRET microscopy

Changes in intracellular Ca^2+^ ([Ca^2+^]_i_) was monitored using Fura-2 ratiometric imaging. Briefly, cells on coverslips were loaded with 1 μM Fura-2 AM for 30 min in an experimental buffer, composed of (in mM): 137 NaCl, 5 KCl, 2 CaCl_2_, 1 MgCl_2_, 10 glucose, and 10 HEPES, pH adjusted to 7.4 with NaOH. The coverslip was then mounted in a perfusion chamber on an inverted microscope (Olympus IX71; Vienna, Austria) with a 20×/0.75 objective and perfused with indicated solutions at 37°C. During the recordings using Live Acquisition v2.6 software (FEI, Planegg, Germany), cells were excited alternately using 340/26 and 380/11 nm filters (Semrock, Rochester, NY, USA) in an Oligochrome excitation system (FEI), and fluorescent images were captured using 510/84-nm emission filter (Semrock) with an ORCA-03G digital CCD camera (Hamamatsu, Herrsching am Ammersee, Germany). The 340/380 ratio was used as an index of cytosolic Ca^2+^ levels.

ER luminal Ca^2+^ levels, [Ca^2+^]_ER_, were monitored in cells transfected with a CFP/YFP FRET-based D1ER sensor at a 535/470 emission ratio using a 430/24-nm excitation filter, 505dcx dichroic, and 470/24- and 535/30-nm emission filters (Chroma Technology, Bellows Falls, VT, USA) with an additional OptoSplit II (Cairn Research, Faversham, UK) in the same setup. Dynamic FRET between STIM1-CFP and YFP-Orai1 was measured using the same setting used for D1ER.

N_FRET_ was performed in the same epifluorescence microscope setting as described previously [31]. Briefly, cells transfected with indicated CFP/YFP fusion constructs were visualized with a 60×/1.42 oil objective using 430/24- and 500/20-nm excitation filters (Chroma Technology). CFP, YFP, and FRET images with 1 s exposure were captured using 505dcx dichroic and 470/24- and 535/30-nm emission filters (Chroma Technology) in the OptoSplit II (Cairn Research). Using ImageJ 1.51n software, images were registered to ensure accurate pixel alignment in all images. N_FRET_ was then calculated using the pixFRET plugin after background subtraction and correction for donor and acceptor bleedthrough and normalized against donor and acceptor levels [42].

### TIRF microscopy

TIRF microscopy was performed using an Olympus IX81 motorized inverted microscope (Olympus, Waltham, MA, USA) with a TIRF-optimized Olympus Plan APO 60×/1.45 oil immersion objective and Lambda 10–3 filter wheel (Sutter Instruments, Novato, CA, USA) containing 480/40, 540/30, and 575lp emission filters (Chroma Technology), as described previously [29]. CFP, YFP, and mCherry were excited by 445-, 514-, and 561-nm lasers, respectively, via cell^TIRF Control 1.3 software. Images were collected every 5 s using an ORCA-Flash4.0 camera (Hamamatsu, Bridgewater, NJ, USA) and MetaMorph software (Molecular Devices, San Jose, CA, USA). Cells plated on poly-l-lysine–coated glass-bottom dishes (MatTek Corporation, Ashland, MA, USA) were perfused with indicated solutions at 37°C during image acquisition. The size, distribution, and rate of clustering of proteins were analyzed using ImageJ 1.51n.

### Whole-cell patch-clamp recording

HEK293 cells on coverslips were mounted in a perfusion chamber on an inverted microscope (Zeiss Axiovert 200; Jena, Germany) with a 40×/0.75 objective and perfused with bath solution, containing 120 mM NaCl, 2.8 mM KCl, 0.5 or 10 mM CaCl_2_, 1 mM MgCl_2_, 20 mM TEA-Cl, 10 mM glucose, and 10 mM HEPES (pH 7.4 with NaOH). Cells were selected based on visual inspection of the relative fluorescence of coexpressed YFP-STIM1 and Orai1-CFP using an Oligochrome excitation system and Oligocon v1.1.14 software (FEI). For passive store depletion, the patch pipette was pulled using a P-1000 micropipette puller (Sutter Instruments) and filled with the intracellular solution containing 130 mM K-gluconate, 8 mM NaCl, 5 mM MgCl_2_, 10 mM HEPES, and 20 mM EGTA (pH 7.2 with KOH) and that had a resistance of 2–3 MΩ. Patch-clamp recordings were performed in whole-cell configuration using an Axopatch 200B amplifier and Clampex v11.0.1 software (Molecular Devices) at 22°C–25°C. An Ag/AgCl electrode was used as a reference. Pipette capacitance was compensated before recordings. The current was recorded during voltage ramps ranging from −90 to +90 mV over 1 s, applied every 5 s from a holding potential of 0 mV, filtered at 2 kHz, and digitized at 10 kHz using Digidata 1440A (Molecular Devices). In Clampfit v11.0.1, the current recorded during the first few ramps was used for leak subtraction of the subsequent current recordings. All voltages were corrected for a liquid junction potential of 13.5 mV between the bath and pipette solutions, and *I*_CRAC_ was measured at −80 mV. Cells with very low expression of either YFP-STIM1 or Orai1-CFP were not selected for analysis since these cells had extremely low *I*_CRAC_.

### Western blot and Co-IP

Cells were washed with 1×PBS and lysed in ice-cold Pierce IP lysis buffer supplemented with protease inhibitor cocktail (Thermo Fisher Scientific). Cell lysates were centrifuged (10,000 × *g*, 10 min at 4°C) and quantified by Pierce BCA protein assay kit (Thermo Fisher Scientific). Co-IP was done using Pierce Anti-c-myc magnetic beads (Thermo Fisher Scientific) as per the manufacturer’s instructions. The immunoprecipitants were eluted in NuPAGE LDS sample buffer (Thermo Fisher Scientific) with 5% DTT by heating at 95°C for 10 min. The isolated tissue was homogenized in lysis buffer (1% Nonidet P-40, 50 mM Tris-HCl, 150 mM NaCl, 2 mM EDTA, 5% glycerol [pH 8]) containing protease inhibitor cocktail tablet with Precellys 24 tissue homogenizer (2 × 20 s, 6,500 rpm) and then was centrifuged for 15 min at 20,000 × *g*. Solubilized proteins from tissue lysates (500 μg, 1 mg/ml) were incubated with 50 μl of washed Protein A agarose (Merck, Vienna, Austria) and gently rotated for 1 h at 4°C to remove nonspecifically bound proteins. Precleared supernatants were incubated overnight with 2 μg of STIM1 antibody (BD Biosciences, San Jose, CA, USA) at 4°C employing an overhead shaker. On the following day, 50 μl of Protein A agarose slurry was added to the lysate and gently rotated for 2 h at 4°C. The beads were washed 5 times with lysis buffer, resuspended in 50 μl of Lämmli buffer, and heated to 95°C for 10 min. The proteins were resolved in NuPAGE 4%–12% Bis-Tris gel (Thermo Fisher Scientific) and transferred to 0.2-μm PVDF membranes using the Trans-Blot Turbo Transfer System (Bio-Rad, Hercules, CA, USA). Membranes were blocked with 5% (w/v) nonfat milk in Tris-buffered saline containing 0.1% Tween 20 (TBST, 25°C, 1 h) and incubated with primary antibodies overnight at 4°C. The following primary antibodies (diluted in 5% [w/v] BSA-TBST) were used: myc (1:1,000, 2276; Cell Signaling Technology, Danvers, MA, USA), STIM1 (1:1,000, 4916; Cell Signaling Technology), Orai1 (1:1,000, produced against C-terminal epitope ELAEFARLQDQLDHRGD and affinity purified by Lofstrand Labs Limited [Gaithersburg, MD, USA]), TMEM38A (1:200, sc-390054; Santa Cruz Biotechnology and 1:1,000, ATC-002; Alomone, Jerusalem, Israel), TMEM38B (1:1,000, PA5-20859; Thermo Fisher Scientific), and β-actin (1:2,500, ab8224; Abcam, Cambridge, MA, USA). After washing, the membranes were incubated with HRP-conjugated goat anti-mouse IgG or goat anti-rabbit IgG (1:10,000, 4% [w/v] nonfat milk-TBST; Jackson Immunoresearch, West Grove, PA, USA) (25°C, 1 h). Immunoreactive bands were visualized using SuperSignal West Pico Chemiluminescent Substrate (Thermo Fisher Scientific) in ChemiDoc MP Imaging System (Bio-Rad), and densitometric evaluation was performed using Image Lab 6.0 software (Bio-Rad).

### RNA isolation and quantitative real-time PCR (qPCR)

RNA was isolated from cell lysates using QIAshredder and RNeasy Mini Kit (Qiagen) and reverse transcribed using a high-capacity cDNA reverse transcription kit (Applied Biosystems, Foster City, CA, USA) in a thermal cycler (Bio-Rad) according to the manufacturer’s protocol. qPCR was performed using QuantiFast SYBR Green RT–PCR kit (Qiagen) in a LightCycler 480 (Roche Diagnostics, Vienna, Austria). Relative expression of the target gene was normalized to mouse *GAPDH* as a reference gene. Primers used for mouse *TRIC-A* mRNA were forward: 5′-CATCACGCACACCACCACTA-3′ and reverse: 5′-TGTTCCACGTTGGACAGGAG-3′ (Eurofins Genomics, Vienna, Austria).

### Statistical analysis

Data analyses were performed using OriginPro 2015 (OriginLab, Northampton, MA, USA) and Prism 5 (GraphPad Software, San Diego, CA, USA). Data are expressed as mean ± SEM. *n* represents the number of cells from at least 3 independent experiments unless specified otherwise. The approximate normal distribution of data was assessed by z-value of skewness and kurtosis and D'Agostino–Pearson omnibus normality test. If normally distributed, unpaired *t* test (2 groups) or one-way ANOVA followed by Dunnett’s multiple comparison test (more than 2 groups) was used to test the statistical significance (with Welch’s correction for significantly different variances between groups); otherwise, Mann–Whitney rank test (2 groups) or Kruskal–Wallis test followed by Dunn’s multiple comparison test (more than 2 groups) was applied. A χ^2^ test was used to analyze responses in cell populations. All tests were two-tailed, and *p* values < 0.05 were considered significant.

## Supporting information

S1 Data Excel spreadsheet containing the underlying numerical data for generating graphs in Figs 1–6, S1–S6 Figs, S8 Fig, and S9 Fig. (XLSX)

S1 Fig TRIC-A modifies frequency and amplitude of RyR2-mediated ER luminal Ca^2+^ oscillations. (A) Representative western blot showing the absence of endogenous TRIC-A bands at approximately 33 kDa using anti-TRIC-A antibody in wild-type (−) and TRIC-A-mCherry–transfected (+) HEK293 cells, *n* = 3 independent experiments. TRIC-A-mCherry and β-actin were used as the positive control and loading control, respectively. (B) Representative epifluorescence images of an HEK293 cell expressing mCerulean-ER-5 (top left, green) or CFP-GPI (bottom left, green) with TRIC-A-mCherry (middle, magenta) along with an overlay (right) of both proteins under basal conditions. Scale bar = 10 μm (C) Traces of [Ca^2+^]_ER_-sensitive D1ER FRET ratio, representing SOICR-associated oscillations in an mCherry-ER-3– (control, black) or TRIC-A-mCherry–transfected (TRIC-A, red) HEK293_RyR2 cell and lack of oscillations in a 3 μM BTP2-incubated (BTP2, blue) control cell. (D) Ca^2+^ oscillation frequency at 0.1, 0.3, and 1 mM [Ca^2+^]_o_ and (E) amplitude at 1 mM [Ca^2+^]_o_ in TRIC-A cells (*n* = 32) versus controls (*n* = 23); *p < 0.05, ***p < 0.001; bars represent mean ± SEM. Underlying data in panels (C–E) are included in S1 Data. BTP2, N-[4-[3,5-Bis(trifluoromethyl)pyrazol-1-yl]phenyl]-4-methylthiadiazole-5-carboxamide; CFP, cyan fluorescent protein; D1ER, genetically encoded ER-targeted Ca^2+^ sensor; ER, endoplasmic reticulum; FRET, Förster resonance energy transfer; GPI, glycosylphosphatidylinositol; HEK293, human embryonic kidney 293; RyR, ryanodine receptor; SOICR, store-overload–induced Ca^2+^ release; TRIC, trimeric intracellular cation.
(TIF)

S2 Fig 2-APB and Orai1-E106Q reduces the frequency of RyR2-mediated cytosolic Ca^2+^ oscillations. Traces of cytosolic Ca^2+^-sensitive Fura-2 ratio represent SOICR-associated oscillations in a (A) control (black) or 2-APB–incubated (red) cell and a (C) YFP (black) or YFP-Orai1-E106Q (red) transfected cell. Bars show mean ± SEM values for Ca^2+^ oscillation frequency at 0.1, 0.3, and 1 mM [Ca^2+^]_o_ in (B) 2-APB–incubated cells (*n* = 98) versus controls (*n* = 81) and (D) YFP-Orai1-E106Q-transfected cells (*n* = 43) versus YFP-transfected controls (*n* = 23), ***p < 0.001. Underlying data in panels (A–D) are included in S1 Data. Fura-2, cytosolic Ca^2+^-sensitive fluorescent indicator; Orai1, Ca^2+^-release–activated Ca^2+^ channel 1; RyR, ryanodine receptor; SOICR, store-overload–induced Ca^2+^ release; YFP, yellow fluorescent protein; 2-APB, 2-Aminoethoxydiphenylborane.
(TIF)

S3 Fig TRIC-A moderately attenuates SOCE upon BHQ-mediated store depletion in HEK293_RYR2 cells and RBL-2H3 cells. Average cytosolic Ca^2+^-sensitive Fura-2 traces in mCherry-ER-3 (control, black) or TRIC-A-mCherry (TRIC-A, red)–transfected (A) HEK293_RyR2 cells and (E) RBL-2H3 cells, showing SOCE after ER Ca^2+^ depletion with 30 μM BHQ. Bar graphs show mean ± SEM values for (B, F) SOCE rate, (C, G) peak and sustained SOCE amplitude, and (D) ER Ca^2+^ release peak amplitude in TRIC-A (+) (*n* = 52) versus control (*n* = 54) HEK293_RyR2 cells and TRIC-A (+) (*n* = 25) versus control (*n* = 27) RBL-2H3 cells; *p < 0.05, **p < 0.01, ***p < 0.001. Underlying data in panels (A–G) are included in S1 Data. BHQ, 2,5-Di-*t*-butyl-1,4-benzohydroquinone; ER, endoplasmic reticulum; Fura-2, cytosolic Ca^2+^-sensitive fluorescent indicator; HEK293, human embryonic kidney 293; ns, nonsignificant; RBL-2H3, rat basophilic leukemia cell line; RyR, ryanodine receptor; SOCE, store-operated Ca^2+^ entry; TRIC, trimeric intracellular cation.
(TIF)

S4 Fig TRIC-A knockdown promotes SOCE in HL-1 cells. (A) Bars show mRNA expression of *TRIC-A* in HL-1 cells transfected with si-scr or si-TRIC-A and normalized to the housekeeping gene *GAPDH*. *n* = 3 independent experiments. (B) Traces of cytosolic Ca^2+^-sensitive Fura-2 ratio in HL-1 cells, transfected with si-scr or si-TRIC-A, showing SOCE after SR Ca^2+^ depletion with 10 mM caffeine + 30 μM BHQ. Bars show (C) SOCE rate and (D) peak and sustained SOCE amplitude in si-TRIC-A (*n* = 361) versus si-scr (*n* = 375) transfected cells, *p < 0.05. Underlying data in panels (A–D) are included in S1 Data. BHQ, 2,5-Di-*t*-butyl-1,4-benzohydroquinone; Fura-2, cytosolic Ca^2+^-sensitive fluorescent indicator; HL-1, mouse atrial muscle cell line; si-scr, scrambled small interfering RNA; SOCE, store-operated Ca^2+^ entry; SR, sarcoplasmic reticulum; TRIC, trimeric intracellular cation.
(TIF)

S5 Fig TRIC-A does not alter CCh-induced Ca^2+^ depletion from IP_3_R stores and thapsigargin-mediated SOCE in HEK293 cells. (A) Average cytosolic Ca^2+^-sensitive Fura-2 traces in mCherry-ER3 (control, black) or TRIC-A-mCherry (TRIC-A, red)–transfected HEK293 cells, showing 100 μM CCh-induced Ca^2+^ depletion from IP_3_R stores. (B) Bar graphs show peak amplitude of IP_3_R store-Ca^2+^ release in TRIC-A (+) (*n* = 54) versus control (*n* = 79) HEK293 cells. (C) Average cytosolic Ca^2+^-sensitive Fura-2 traces in mCherry-ER-3 (control, black) or TRIC-A-mCherry (TRIC-A, red)–transfected HEK293 cells, showing SOCE after ER Ca^2+^ depletion with 1 μM thapsigargin. Bar graphs show (D) ER Ca^2+^ release peak amplitude, (E) SOCE rate, and (F) peak and sustained SOCE amplitude in TRIC-A (+) (*n* = 96) versus control (*n* = 116) HEK293 cells. *p < 0.05; mean values ± SEM are shown. Underlying data in panels (A–F) are included in S1 Data. CCh, carbachol; ER, endoplasmic reticulum; Fura-2, cytosolic Ca^2+^-sensitive fluorescent indicator; HEK293, human embryonic kidney 293; IP_3_R, inositol 1,4,5-triphosphate receptor; ns, nonsignificant; SOCE, store-operated Ca^2+^ entry; TRIC, trimeric intracellular cation.
(TIF)

S6 Fig TRIC-A does not affect endogenous and transient overexpression of STIM1 and Orai1 in HEK293 cells. (A) Representative western blots for STIM1, Orai1, and TRIC-A-mCherry expression in control and TRIC-A-mCherry–transfected HEK293 cells with β-actin as a loading control, *n* = 6 independent experiments. (B) Densitometric evaluation of immunoreactive bands of Orai1 and STIM1 shown in (A). (C) Epifluorescence of overexpressed Orai1-CFP and YFP-STIM1 in HEK293 cells coexpressing mCherry-ER-3 (control) or TRIC-A-mCherry (TRIC-A), *n* = 18 in each group. Bars represent mean ± SEM. Underlying data in panels B and C are included in S1 Data. CFP, cyan fluorescent protein; HEK293, human embryonic kidney 293; ns, nonsignificant; Orai1, Ca^2+^-release–activated Ca^2+^ channel 1; STIM1, stromal interaction molecule 1; TRIC, trimeric intracellular cation; YFP, yellow fluorescent protein.
(TIF)

S7 Fig STIM1 coimmunoprecipitates with TRIC-A in HEK293 cells. Representative Co-IP of endogenous STIM1 with myc-TRIC-A expressed in HEK293 cells. Lysates were obtained from basal (−) and ER-depleted (+) (100 μM CCh + 30 μM BHQ) HEK293 cells, n = 3 independent experiments. BHQ, 2,5-Di-*t*-butyl-1,4-benzohydroquinone; CCh, carbachol; Co-IP, coimmunoprecipitation; HEK293, human embryonic kidney 293; STIM1, stromal interaction molecule 1; TRIC, trimeric intracellular cation.
(TIF)

S8 Fig TRIC-A coclusters with STIM1 EF-hand mutant independent of store depletion in HEK293 cells, which lack SOICR-induced Ca^2+^ oscillations irrespective of STIM1 EF-hand mutant expression. (A) Representative TIRF images of a basal (top) and ER-depleted (100 μM CCh + 30 μM BHQ) (bottom) HEK293 cell expressing YFP-STIM1-D76A (left, green) and TRIC-A-mCherry (middle, magenta) with an overlay (right) of both proteins. Scale bar = 5 μm. (B) Line scans of YFP-STIM1-D76A and TRIC-A-mCherry in a basal and ER-depleted cell shown in (A). (C) Bars show mean ± SEM values of Mander’s coefficient for proportion of TRIC-A-mCherry colocalized with STIM1 under basal and ER-depleted conditions, *n* = 8 from 3 different experiments. (D) Traces of cytosolic Ca^2+^-sensitive Fura-2 ratio, represent constitutive Ca^2+^ influx, leading to sustained cytosolic Ca^2+^ rise, but not oscillations, in HEK293 cells expressing YFP-STIM1-D76A (green, *n* = 98) and lack of any constitutive Ca^2+^ influx and oscillations in HEK293 cells (black, *n* = 45); mean ± SEM values are shown. Underlying data in panels (B–D) are included in S1 Data. BHQ, 2,5-Di-*t*-butyl-1,4-benzohydroquinone; CCh, carbachol; ER, endoplasmic reticulum; Fura-2, cytosolic Ca^2+^-sensitive fluorescent indicator; HEK293, human embryonic kidney 293; ns, nonsignificant; SOICR, store-overload–induced Ca^2+^ release; STIM1, stromal interaction molecule 1; TIRF, total internal reflection fluorescence; TRIC, trimeric intracellular cation; YFP, yellow fluorescent protein.
(TIF)

S9 Fig TRIC-A alters the puncta size distribution of Orai1 and STIM1 upon store depletion. Proportion of punctae (%) of various sizes of (A) Orai1-CFP (number of punctae = 4,462 versus 4,836) and (B) YFP-STIM1 (number of punctae = 4,357 versus 4,519) in control and TRIC-A (+) cells (*n* = 18 each). Overall size distribution in TRIC-A cells was significantly different from that in controls (***p < 0.001, χ^2^ test). (C) Mean ± SEM of puncta density (number/μm^2^) of Orai1-CFP and YFP-STIM1 in TRIC-A (+) cells compared to controls. Underlying data in panels (A–C) are included in S1 Data. CFP, cyan fluorescent protein; ns, nonsignificant; Orai1, Ca^2+^-release–activated Ca^2+^ channel 1; STIM1, stromal interaction molecule 1; TRIC, trimeric intracellular cation; YFP, yellow fluorescent protein.
(TIF)

S1 Video **Time-lapse TIRF image stack showing clustering of YFP-STIM1 (left) and TRIC-A-mCherry (right) coexpressed in HEK293 cell upon ER Ca**^**2+**^ **depletion, related to Fig 5.** ER, endoplasmic reticulum; HEK293, human embryonic kidney 293; STIM1, stromal interaction molecule 1, TIRF, total internal reflection fluorescence; TRIC, trimeric intracellular cation; YFP, yellow fluorescent protein.
(MP4)

S2 Video Time-lapse TIRF image stack showing the absence of clustering of TRIC-A-mCherry expressed in HEK293 cell upon ER Ca^2+^ depletion, related to Fig 5. ER, endoplasmic reticulum, HEK293, human embryonic kidney 293; TIRF, total internal reflection fluorescence; TRIC, trimeric intracellular cation.
(MP4)

S1 Raw Images Original, uncropped images supporting blot results reported in Fig 5, S1 Fig, S6 Fig, and S7 Fig. (PDF)

## Abbreviations

BHQ: 2,5-Di-*t*-butyl-1,4-benzohydroquinone
BTP2: N-[4-[3,5-Bis(trifluoromethyl)pyrazol-1-yl]phenyl]-4-methylthiadizole-5-carboxamide
CCh: carbachol
CFP: cyan fluorescent protein
Co-IP: coimmunoprecipitation
D1ER: genetically encoded ER-targeted Ca^2+^ sensor
ER: endoplasmic reticulum
FRET: Förster resonance energy transfer
Fura-2: cytosolic Ca^2+^-sensitive fluorescent indicator
GPI: glycosylphosphatidylinositol
HEK293: human embryonic kidney 293
HL-1: mouse atrial muscle cell line
*I*_CRAC_: Ca^2+^ release-activated Ca^2+^ current
IP_3_R: inositol 1,4,5-triphosphate receptor
I–V: current–voltage
N_FRET_: normalized FRET
Orai1: Ca^2+^-release–activated Ca^2+^ channel 1
PM: plasma membrane
qPCR: quantitative real-time PCR
RBL-2H3: rat basophilic leukemia cell line
RyR: ryanodine receptor
SARAF: SOCE-associated regulatory factor
SERCA: sarco/endoplasmic reticulum Ca^2+^-ATPase
siRNA: small interfering RNA
si-scr: scrambled siRNA
SOCE: store-operated Ca^2+^ entry
SOICR: store-overload–induced Ca^2+^ release
SR: sarcoplasmic reticulum
STIM1: stromal interaction molecule 1
TIRF: total internal reflection fluorescence
TMEM: transmembrane protein
TRIC channel: trimeric intracellular cation channel
YFP: yellow fluorescent protein
2-APB: 2-Aminoethoxydiphenylborane
