# Supplementary figures and images for: TRIC-A shapes oscillatory Ca2+ signals by interaction with STIM1/Orai1 complexes
Source: PLoS Biol. 2020 Apr 24;18(4):e3000700. doi: 10.1371/journal.pbio.3000700 (PMC7202670; doi:10.1371/journal.pbio.3000700)

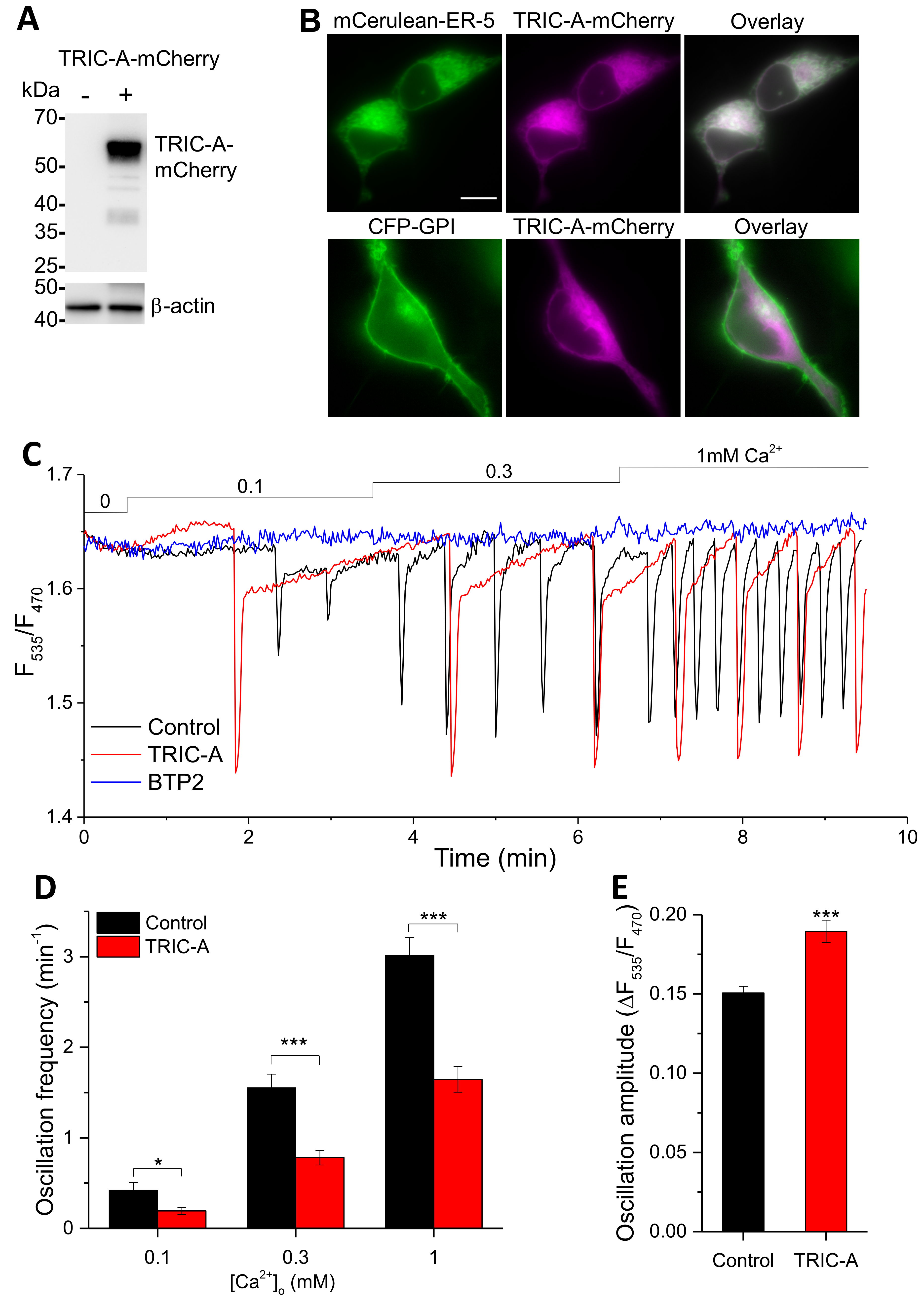

Supplement: S1 Fig — (A) Representative western blot showing the absence of endogenous TRIC-A bands at approximately 33 kDa using anti-TRIC-A antibody in wild-type (−) and TRIC-A-mCherry–transfected (+) HEK293 cells, n = 3 independent experiments. TRIC-A-mCherry and β-actin were used as the positive control and loading control, respectively. (B) Representative epifluorescence images of an HEK293 cell expressing mCerulean-ER-5 (top left, green) or CFP-GPI (bottom left, green) with TRIC-A-mCherry (middle, magenta) along with an overlay (right) of both proteins under basal conditions. Scale bar = 10 μm (C) Traces of [Ca2+]ER-sensitive D1ER FRET ratio, representing SOICR-associated oscillations in an mCherry-ER-3– (control, black) or TRIC-A-mCherry–transfected (TRIC-A, red) HEK293_RyR2 cell and lack of oscillations in a 3 μM BTP2-incubated (BTP2, blue) control cell. (D) Ca2+ oscillation frequency at 0.1, 0.3, and 1 mM [Ca2+]o and (E) amplitude at 1 mM [Ca2+]o in TRIC-A cells (n = 32) versus controls (n = 23); *p < 0.05, ***p < 0.001; bars represent mean ± SEM. Underlying data in panels (C–E) are included in S1 Data. BTP2, N-[4-[3,5-Bis(trifluoromethyl)pyrazol-1-yl]phenyl]-4-methylthiadiazole-5-carboxamide; CFP, cyan fluorescent protein; D1ER, genetically encoded ER-targeted Ca2+ sensor; ER, endoplasmic reticulum; FRET, Förster resonance energy transfer; GPI, glycosylphosphatidylinositol; HEK293, human embryonic kidney 293; RyR, ryanodine receptor; SOICR, store-overload–induced Ca2+ release; TRIC, trimeric intracellular cation. (TIF) [file pbio.3000700.s002.tif]

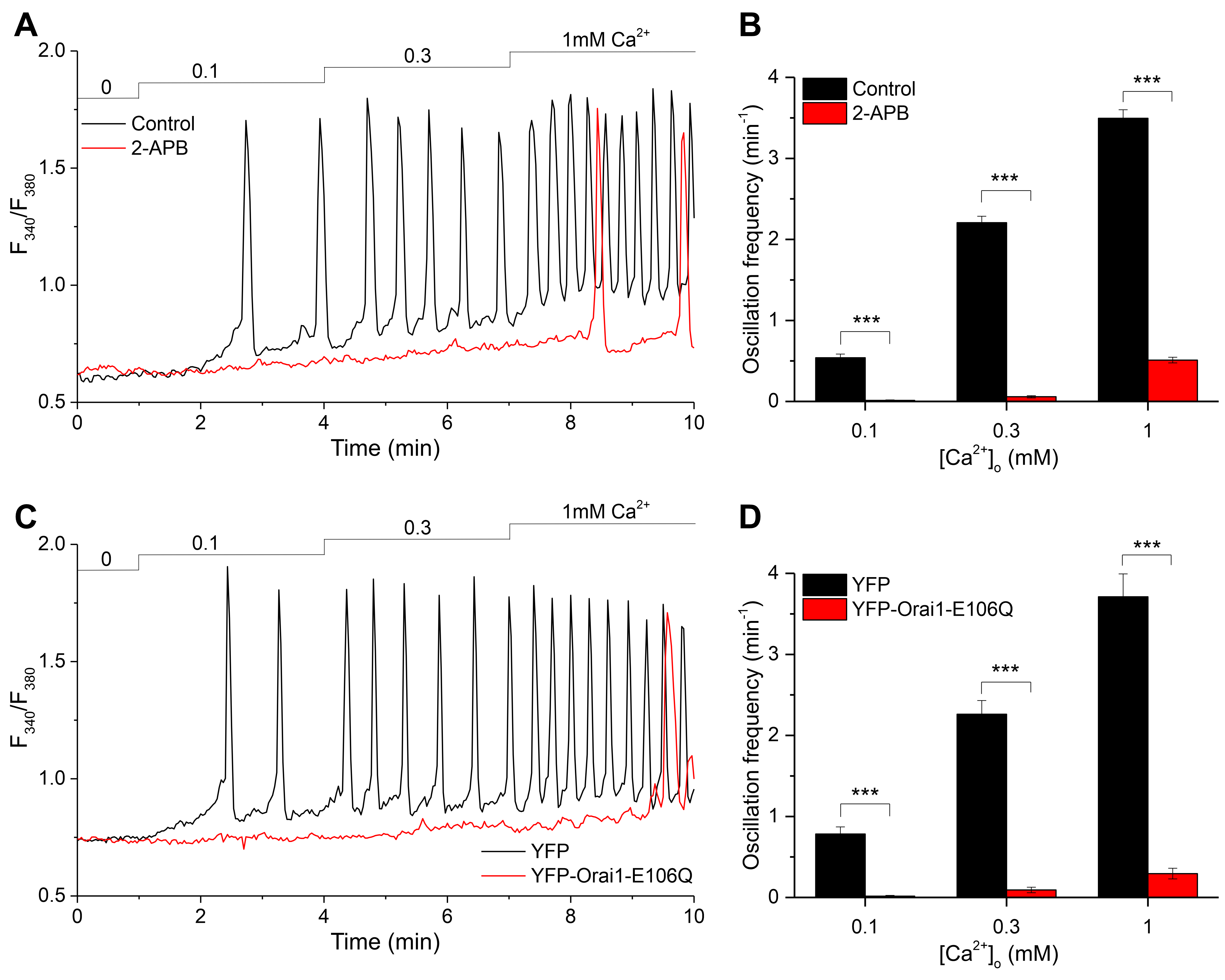

Supplement: S2 Fig — Traces of cytosolic Ca2+-sensitive Fura-2 ratio represent SOICR-associated oscillations in a (A) control (black) or 2-APB–incubated (red) cell and a (C) YFP (black) or YFP-Orai1-E106Q (red) transfected cell. Bars show mean ± SEM values for Ca2+ oscillation frequency at 0.1, 0.3, and 1 mM [Ca2+]o in (B) 2-APB–incubated cells (n = 98) versus controls (n = 81) and (D) YFP-Orai1-E106Q-transfected cells (n = 43) versus YFP-transfected controls (n = 23), ***p < 0.001. Underlying data in panels (A–D) are included in S1 Data. Fura-2, cytosolic Ca2+-sensitive fluorescent indicator; Orai1, Ca2+-release–activated Ca2+ channel 1; RyR, ryanodine receptor; SOICR, store-overload–induced Ca2+ release; YFP, yellow fluorescent protein; 2-APB, 2-Aminoethoxydiphenylborane. (TIF) [file pbio.3000700.s003.tif]

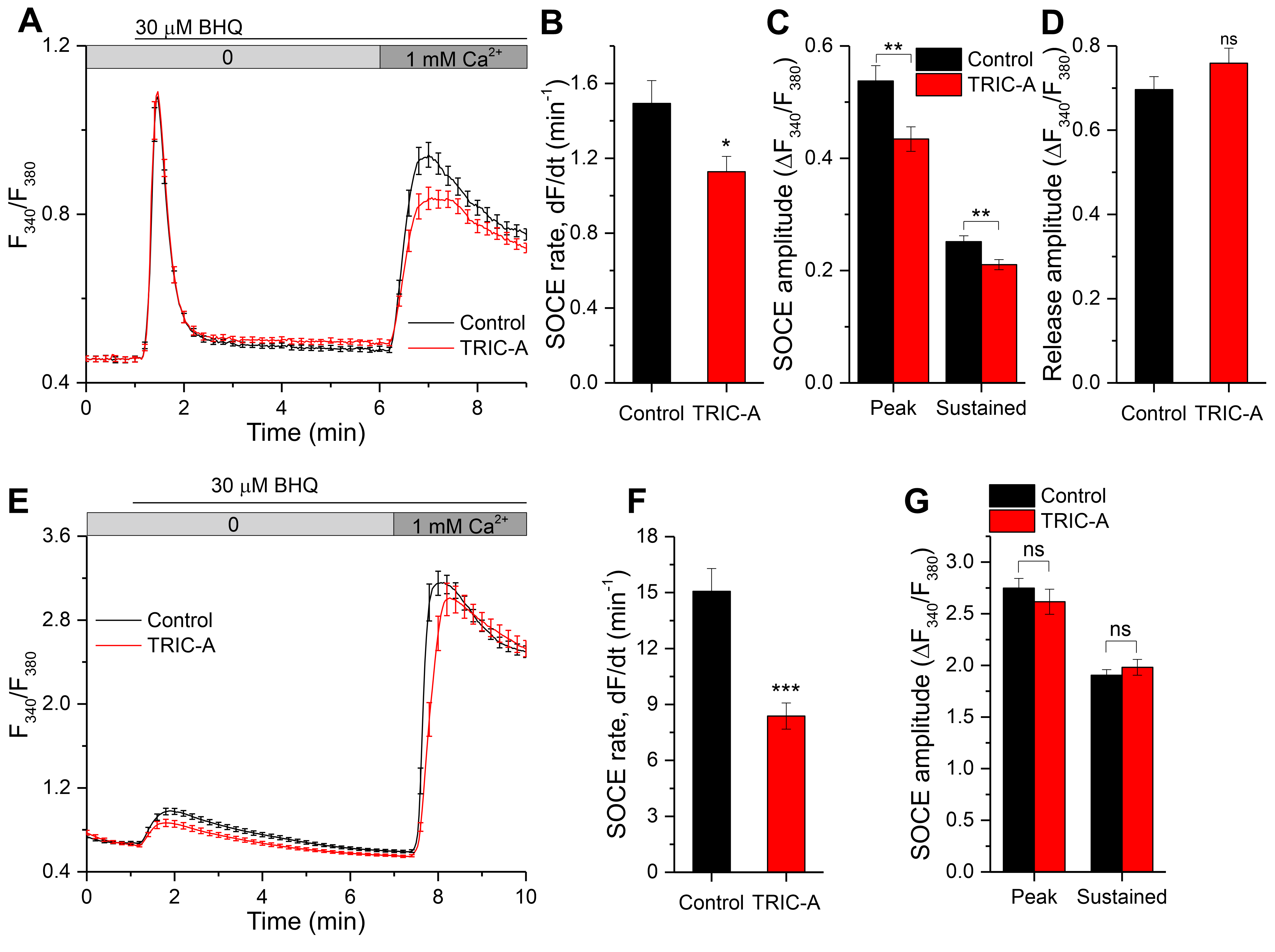

Supplement: S3 Fig — Average cytosolic Ca2+-sensitive Fura-2 traces in mCherry-ER-3 (control, black) or TRIC-A-mCherry (TRIC-A, red)–transfected (A) HEK293_RyR2 cells and (E) RBL-2H3 cells, showing SOCE after ER Ca2+ depletion with 30 μM BHQ. Bar graphs show mean ± SEM values for (B, F) SOCE rate, (C, G) peak and sustained SOCE amplitude, and (D) ER Ca2+ release peak amplitude in TRIC-A (+) (n = 52) versus control (n = 54) HEK293_RyR2 cells and TRIC-A (+) (n = 25) versus control (n = 27) RBL-2H3 cells; *p < 0.05, **p < 0.01, ***p < 0.001. Underlying data in panels (A–G) are included in S1 Data. BHQ, 2,5-Di-t-butyl-1,4-benzohydroquinone; ER, endoplasmic reticulum; Fura-2, cytosolic Ca2+-sensitive fluorescent indicator; HEK293, human embryonic kidney 293; ns, nonsignificant; RBL-2H3, rat basophilic leukemia cell line; RyR, ryanodine receptor; SOCE, store-operated Ca2+ entry; TRIC, trimeric intracellular cation. (TIF) [file pbio.3000700.s004.tif]

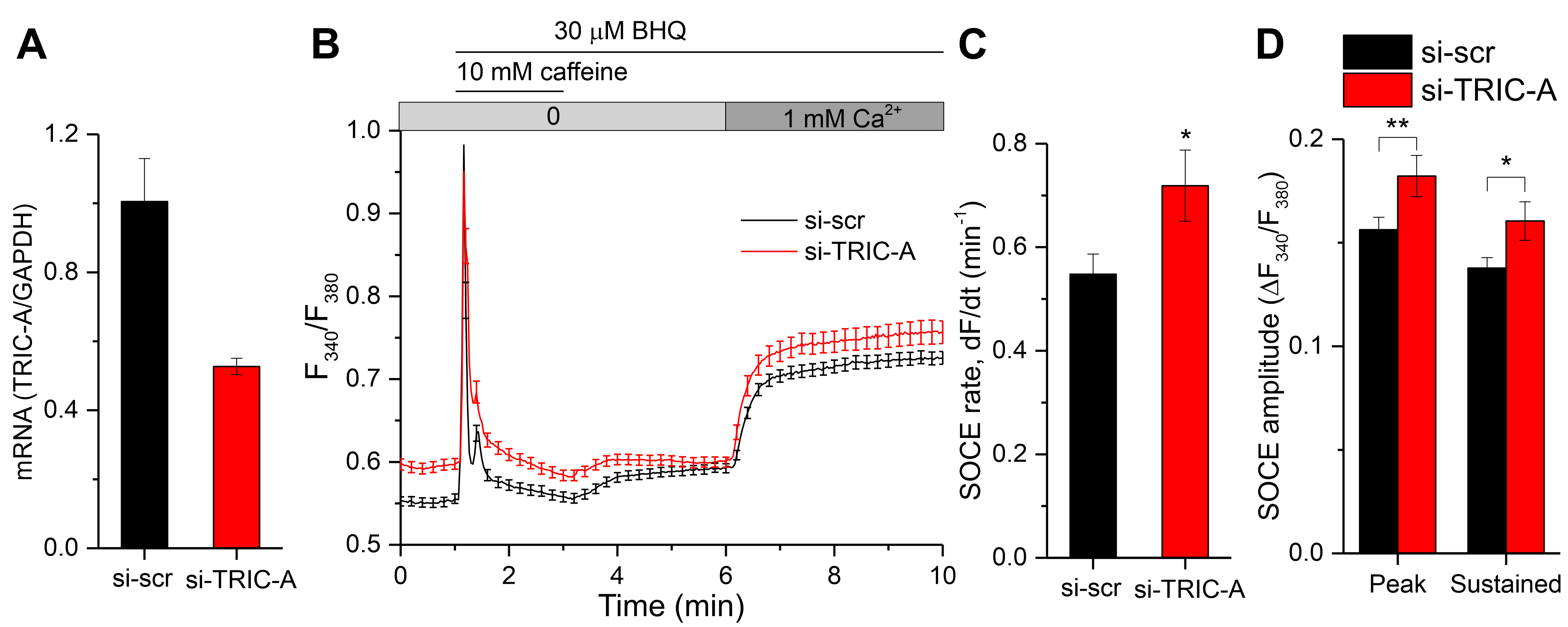

Supplement: S4 Fig — (A) Bars show mRNA expression of TRIC-A in HL-1 cells transfected with si-scr or si-TRIC-A and normalized to the housekeeping gene GAPDH. n = 3 independent experiments. (B) Traces of cytosolic Ca2+-sensitive Fura-2 ratio in HL-1 cells, transfected with si-scr or si-TRIC-A, showing SOCE after SR Ca2+ depletion with 10 mM caffeine + 30 μM BHQ. Bars show (C) SOCE rate and (D) peak and sustained SOCE amplitude in si-TRIC-A (n = 361) versus si-scr (n = 375) transfected cells, *p < 0.05. Underlying data in panels (A–D) are included in S1 Data. BHQ, 2,5-Di-t-butyl-1,4-benzohydroquinone; Fura-2, cytosolic Ca2+-sensitive fluorescent indicator; HL-1, mouse atrial muscle cell line; si-scr, scrambled small interfering RNA; SOCE, store-operated Ca2+ entry; SR, sarcoplasmic reticulum; TRIC, trimeric intracellular cation. (TIF) [file pbio.3000700.s005.tif]

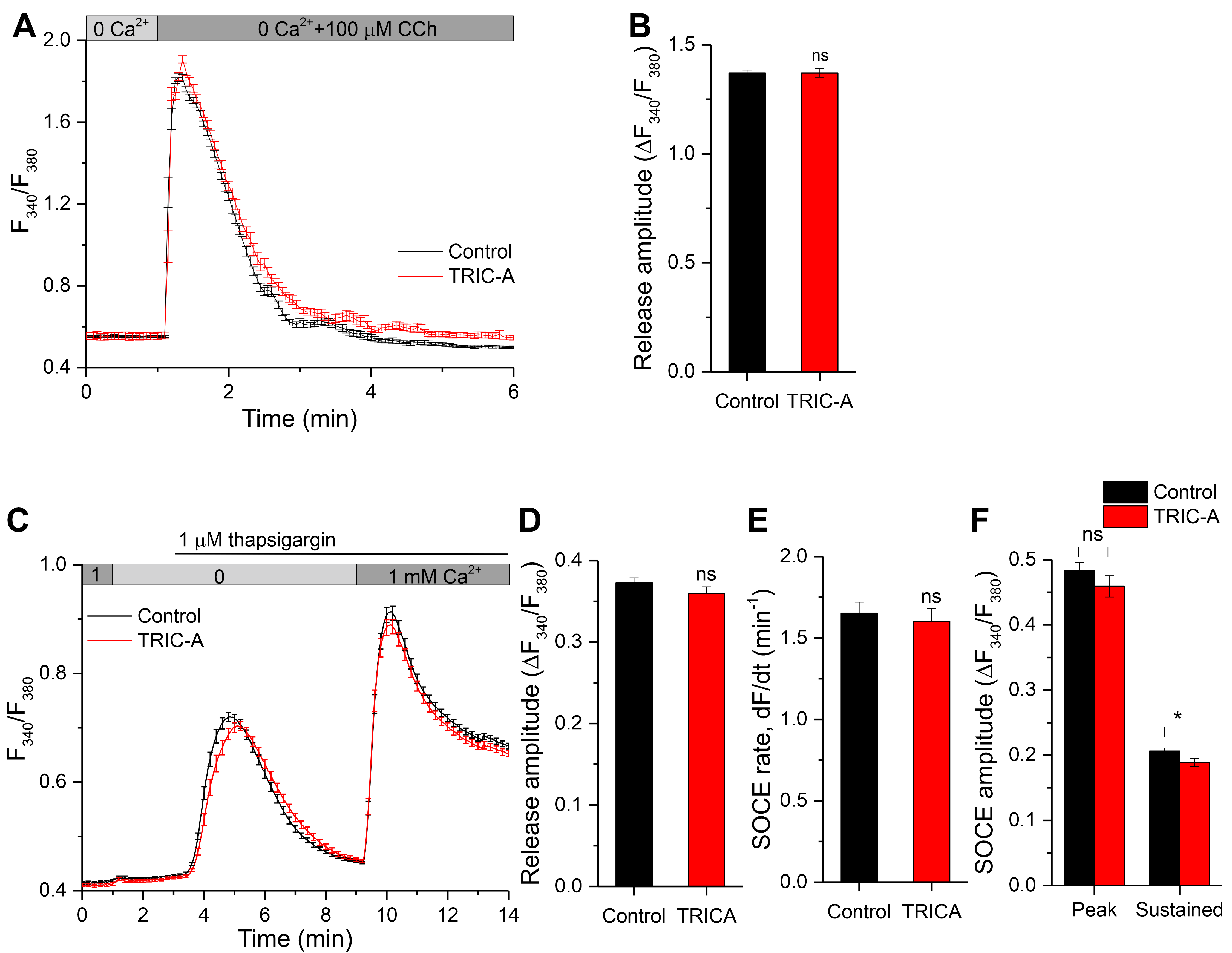

Supplement: S5 Fig — (A) Average cytosolic Ca2+-sensitive Fura-2 traces in mCherry-ER3 (control, black) or TRIC-A-mCherry (TRIC-A, red)–transfected HEK293 cells, showing 100 μM CCh-induced Ca2+ depletion from IP3R stores. (B) Bar graphs show peak amplitude of IP3R store-Ca2+ release in TRIC-A (+) (n = 54) versus control (n = 79) HEK293 cells. (C) Average cytosolic Ca2+-sensitive Fura-2 traces in mCherry-ER-3 (control, black) or TRIC-A-mCherry (TRIC-A, red)–transfected HEK293 cells, showing SOCE after ER Ca2+ depletion with 1 μM thapsigargin. Bar graphs show (D) ER Ca2+ release peak amplitude, (E) SOCE rate, and (F) peak and sustained SOCE amplitude in TRIC-A (+) (n = 96) versus control (n = 116) HEK293 cells. *p < 0.05; mean values ± SEM are shown. Underlying data in panels (A–F) are included in S1 Data. CCh, carbachol; ER, endoplasmic reticulum; Fura-2, cytosolic Ca2+-sensitive fluorescent indicator; HEK293, human embryonic kidney 293; IP3R, inositol 1,4,5-triphosphate receptor; ns, nonsignificant; SOCE, store-operated Ca2+ entry; TRIC, trimeric intracellular cation. (TIF) [file pbio.3000700.s006.tif]

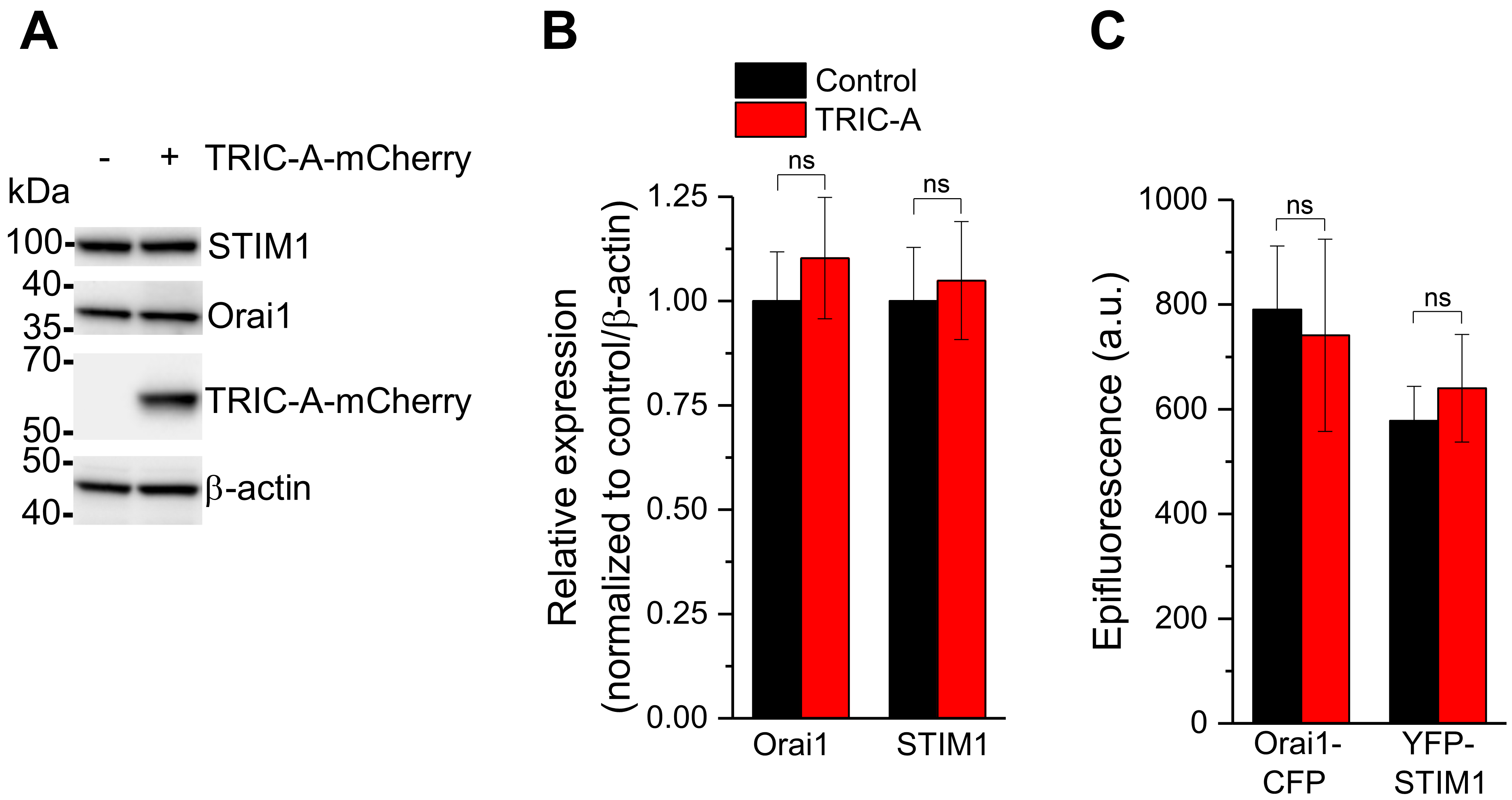

Supplement: S6 Fig — (A) Representative western blots for STIM1, Orai1, and TRIC-A-mCherry expression in control and TRIC-A-mCherry–transfected HEK293 cells with β-actin as a loading control, n = 6 independent experiments. (B) Densitometric evaluation of immunoreactive bands of Orai1 and STIM1 shown in (A). (C) Epifluorescence of overexpressed Orai1-CFP and YFP-STIM1 in HEK293 cells coexpressing mCherry-ER-3 (control) or TRIC-A-mCherry (TRIC-A), n = 18 in each group. Bars represent mean ± SEM. Underlying data in panels B and C are included in S1 Data. CFP, cyan fluorescent protein; HEK293, human embryonic kidney 293; ns, nonsignificant; Orai1, Ca2+-release–activated Ca2+ channel 1; STIM1, stromal interaction molecule 1; TRIC, trimeric intracellular cation; YFP, yellow fluorescent protein. (TIF) [file pbio.3000700.s007.tif]

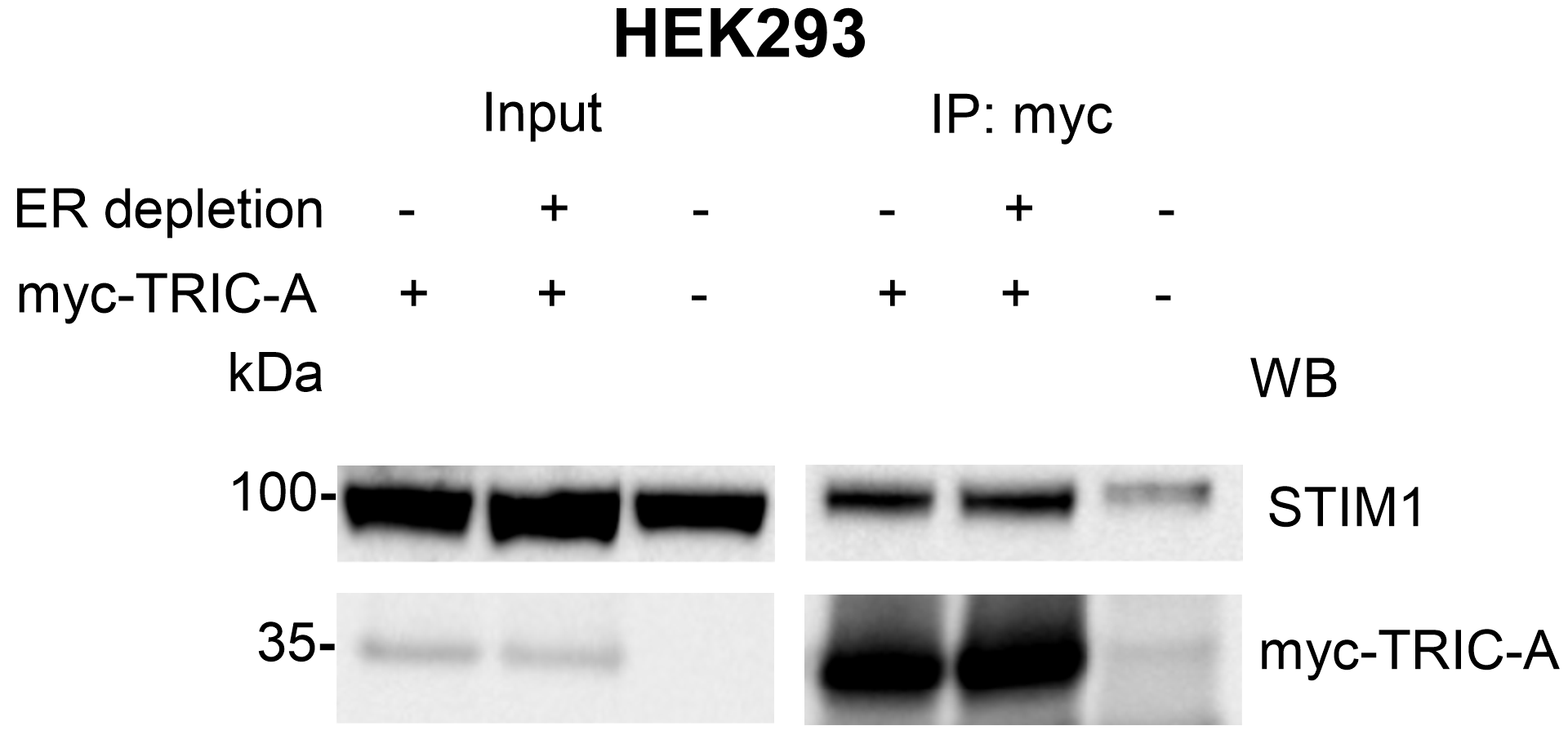

Supplement: S7 Fig — Representative Co-IP of endogenous STIM1 with myc-TRIC-A expressed in HEK293 cells. Lysates were obtained from basal (−) and ER-depleted (+) (100 μM CCh + 30 μM BHQ) HEK293 cells, n = 3 independent experiments. BHQ, 2,5-Di-t-butyl-1,4-benzohydroquinone; CCh, carbachol; Co-IP, coimmunoprecipitation; HEK293, human embryonic kidney 293; STIM1, stromal interaction molecule 1; TRIC, trimeric intracellular cation. (TIF) [file pbio.3000700.s008.tif]

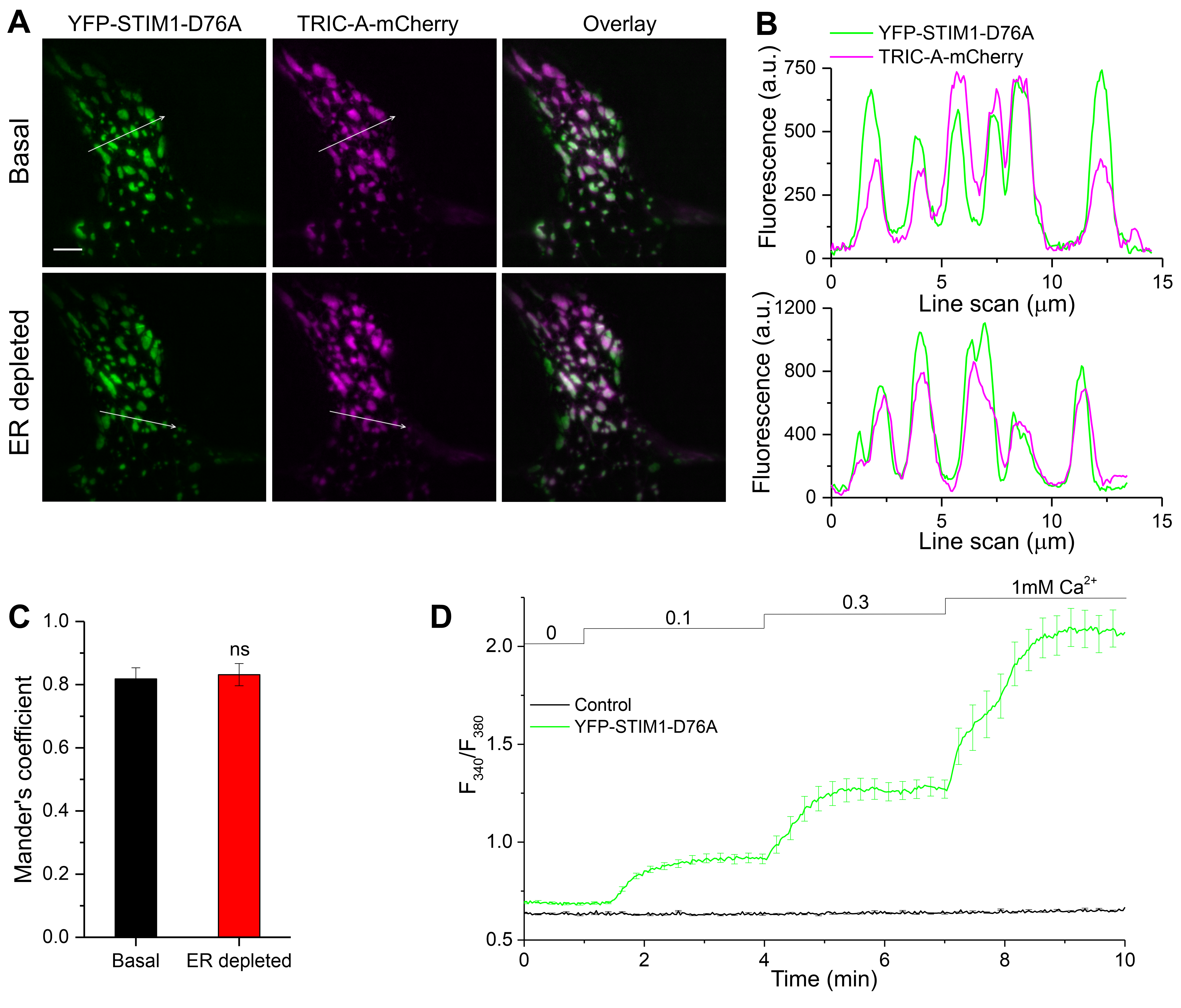

Supplement: S8 Fig — (A) Representative TIRF images of a basal (top) and ER-depleted (100 μM CCh + 30 μM BHQ) (bottom) HEK293 cell expressing YFP-STIM1-D76A (left, green) and TRIC-A-mCherry (middle, magenta) with an overlay (right) of both proteins. Scale bar = 5 μm. (B) Line scans of YFP-STIM1-D76A and TRIC-A-mCherry in a basal and ER-depleted cell shown in (A). (C) Bars show mean ± SEM values of Mander’s coefficient for proportion of TRIC-A-mCherry colocalized with STIM1 under basal and ER-depleted conditions, n = 8 from 3 different experiments. (D) Traces of cytosolic Ca2+-sensitive Fura-2 ratio, represent constitutive Ca2+ influx, leading to sustained cytosolic Ca2+ rise, but not oscillations, in HEK293 cells expressing YFP-STIM1-D76A (green, n = 98) and lack of any constitutive Ca2+ influx and oscillations in HEK293 cells (black, n = 45); mean ± SEM values are shown. Underlying data in panels (B–D) are included in S1 Data. BHQ, 2,5-Di-t-butyl-1,4-benzohydroquinone; CCh, carbachol; ER, endoplasmic reticulum; Fura-2, cytosolic Ca2+-sensitive fluorescent indicator; HEK293, human embryonic kidney 293; ns, nonsignificant; SOICR, store-overload–induced Ca2+ release; STIM1, stromal interaction molecule 1; TIRF, total internal reflection fluorescence; TRIC, trimeric intracellular cation; YFP, yellow fluorescent protein. (TIF) [file pbio.3000700.s009.tif]

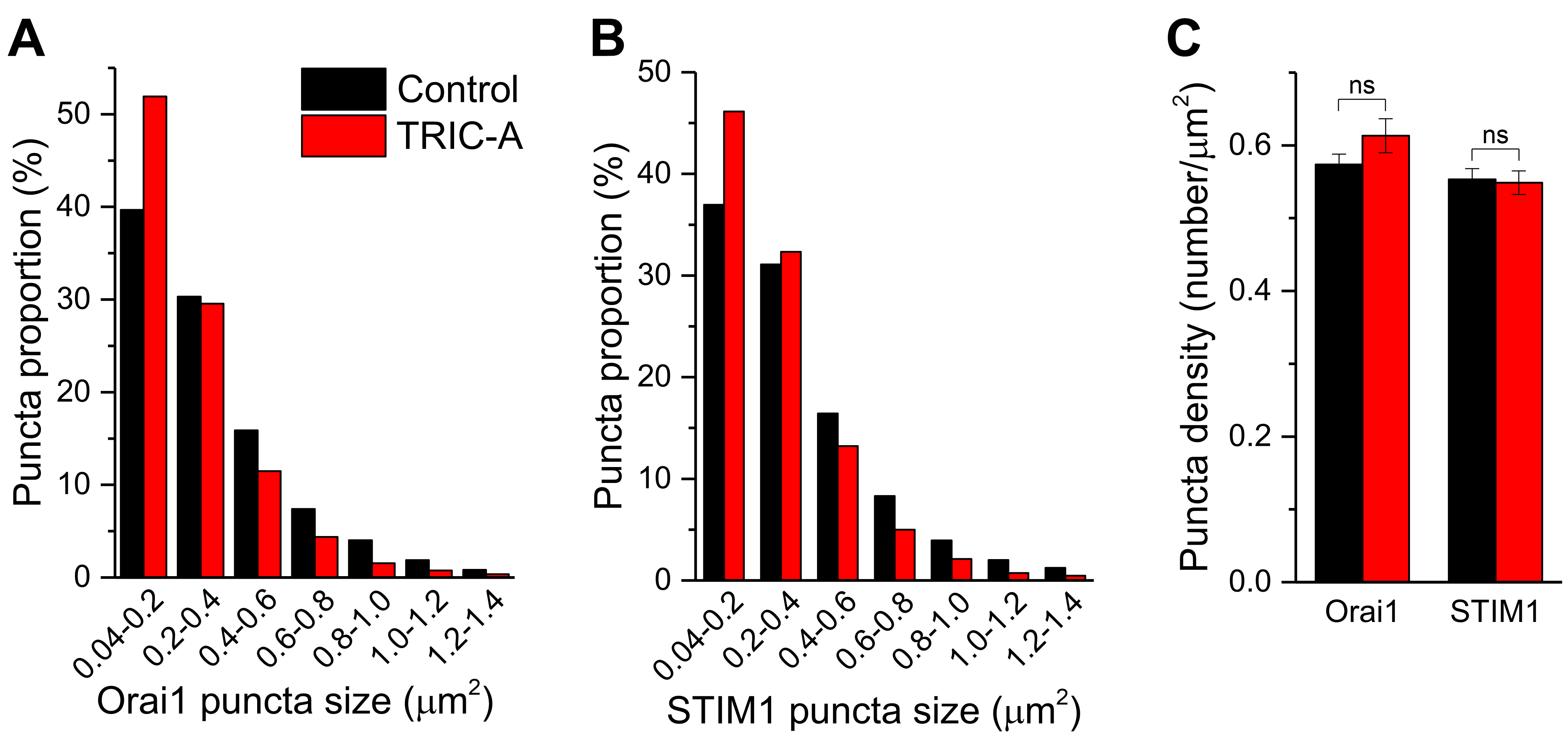

Supplement: S9 Fig — Proportion of punctae (%) of various sizes of (A) Orai1-CFP (number of punctae = 4,462 versus 4,836) and (B) YFP-STIM1 (number of punctae = 4,357 versus 4,519) in control and TRIC-A (+) cells (n = 18 each). Overall size distribution in TRIC-A cells was significantly different from that in controls (***p < 0.001, χ2 test). (C) Mean ± SEM of puncta density (number/μm2) of Orai1-CFP and YFP-STIM1 in TRIC-A (+) cells compared to controls. Underlying data in panels (A–C) are included in S1 Data. CFP, cyan fluorescent protein; ns, nonsignificant; Orai1, Ca2+-release–activated Ca2+ channel 1; STIM1, stromal interaction molecule 1; TRIC, trimeric intracellular cation; YFP, yellow fluorescent protein. (TIF) [file pbio.3000700.s010.tif]

Figure 5

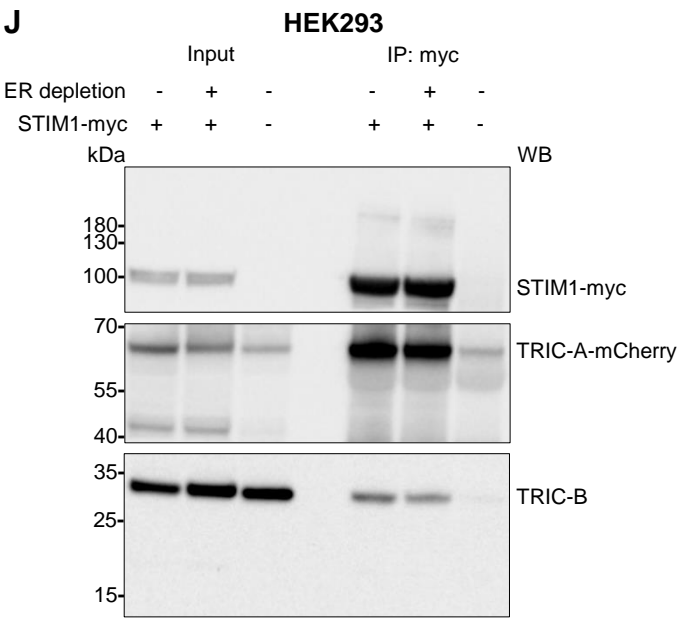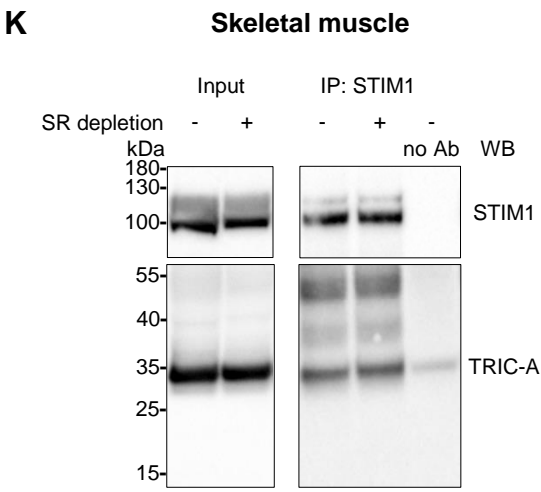

**Figure S1**

**A**

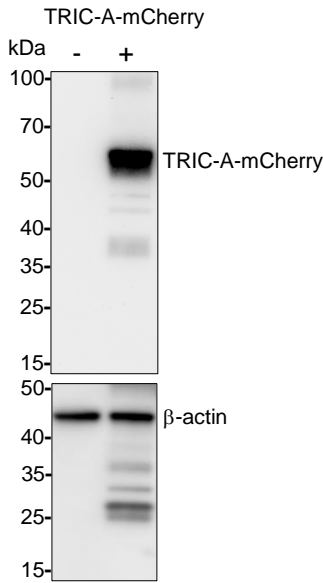

**Figure S6**

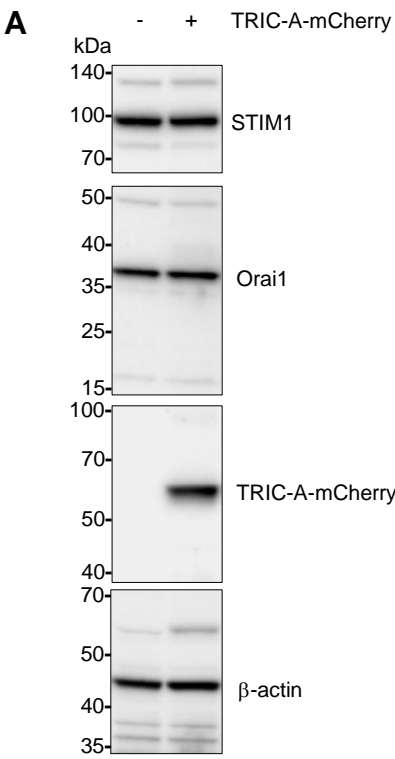

Figure S7

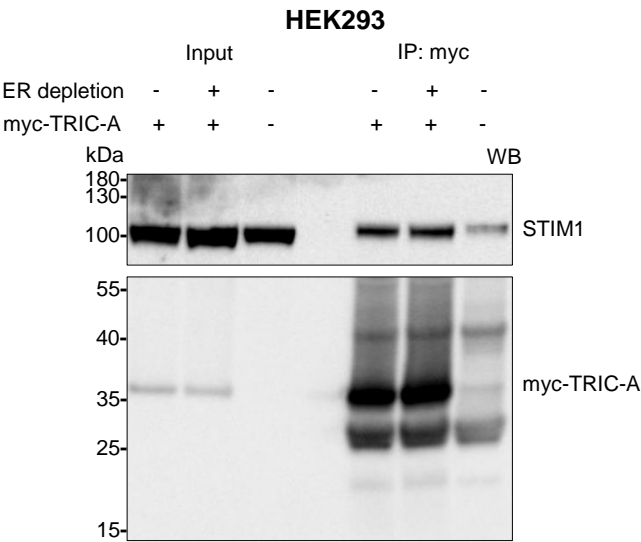

Supplement: S1 Raw Images — (PDF) [file pbio.3000700.s013.pdf]
